# Soil erosion modelling: A global review and statistical analysis

**DOI:** 10.1016/j.scitotenv.2021.146494

**Published:** 2021-08-01

**Authors:** Pasquale Borrelli, Christine Alewell, Pablo Alvarez, Jamil Alexandre Ayach Anache, Jantiene Baartman, Cristiano Ballabio, Nejc Bezak, Marcella Biddoccu, Artemi Cerdà, Devraj Chalise, Songchao Chen, Walter Chen, Anna Maria De Girolamo, Gizaw Desta Gessesse, Detlef Deumlich, Nazzareno Diodato, Nikolaos Efthimiou, Gunay Erpul, Peter Fiener, Michele Freppaz, Francesco Gentile, Andreas Gericke, Nigussie Haregeweyn, Bifeng Hu, Amelie Jeanneau, Konstantinos Kaffas, Mahboobeh Kiani-Harchegani, Ivan Lizaga Villuendas, Changjia Li, Luigi Lombardo, Manuel López-Vicente, Manuel Esteban Lucas-Borja, Michael Märker, Francis Matthews, Chiyuan Miao, Matjaž Mikoš, Sirio Modugno, Markus Möller, Victoria Naipal, Mark Nearing, Stephen Owusu, Dinesh Panday, Edouard Patault, Cristian Valeriu Patriche, Laura Poggio, Raquel Portes, Laura Quijano, Mohammad Reza Rahdari, Mohammed Renima, Giovanni Francesco Ricci, Jesús Rodrigo-Comino, Sergio Saia, Aliakbar Nazari Samani, Calogero Schillaci, Vasileios Syrris, Hyuck Soo Kim, Diogo Noses Spinola, Paulo Tarso Oliveira, Hongfen Teng, Resham Thapa, Konstantinos Vantas, Diana Vieira, Jae E. Yang, Shuiqing Yin, Demetrio Antonio Zema, Guangju Zhao, Panos Panagos

**Affiliations:** a Department of Earth and Environmental Sciences, University of Pavia, Via Ferrata, 1, 27100 Pavia, Italy; b Department of Environmental Sciences, Environmental Geosciences, University of Basel, Basel CH-4056, Switzerland; c Department of Biological Environment, Kangwon National University, Chuncheon 24341, Republic of Korea; d Institute of Geography and Geoecology, Karlsruhe Institute of Technology, Germany; e Faculty of Agricultural Sciences, National University of Loja, Ecuador; f Department of Hydraulics and Sanitation, São Carlos School of Engineering (EESC), University of São Paulo (USP), CxP. 359, São Carlos, SP 13566-590, Brazil; g Federal University of Mato Grosso do Sul, CxP. 549, Campo Grande, MS 79070-900, Brazil; h Soil Physics and Land Management Group, Wageningen University, Wageningen, the Netherlands; i European Commission, Joint Research Centre (JRC), Ispra, Italy; j University of Ljubljana, Faculty of Civil and Geodetic Engineering, Ljubljana, Slovenia; k Institute of Sciences and Technologies for Sustainable Energy and Mobility (STEMS), National Research Council of Italy (CNR), Strada delle Cacce 73, 10135 Torino, Italy; l Soil Erosion and Degradation Research Group, Department of Geography, University of Valencia, Valencia, Spain; m School of Environmental and Rural Science, University of New England, Armidale, NSW 2351, Australia; n INRAE, Unité InfoSol, Orléans 45075, France; o Department of Civil Engineering, National Taipei University of Technology, Taiwan; p Water Research Institute, National Research Council, Bari, Italy; q International Crops Research Institute for the Semi-Arid Tropics (ICRISAT), Ethiopia; r Leibniz-Center for Agricultural Landscape Research Muencheberg (ZALF), Germany; s Met European Research Observatory—International Affiliates Program of the University Corporation for Atmospheric Research, Via Monte Pino snc, 82100 Benevento, Italy; t Faculty of Environmental Sciences, Czech University of Life Sciences Prague, Kamýcká 129, Praha, Suchdol 165 00, Czech Republic; u Department of Soil Science and Plant Nutrition, Faculty of Agriculture, University of Ankara, 06110, Diskapi, Ankara, Turkey; v Water and Soil Resources Research Group, Institute of Geography, Universität Augsburg, Alter Postweg 118, 86159 Augsburg, Germany; w University of Turin, Department of Agricultural, Forest and Food Sciences, Largo Paolo Braccini, 2, 10095 Grugliasco, Italy; x University of Bari Aldo Moro, Department of Agricultural and Environmental Sciences, Bari, Italy; y Leibniz-Institute of Freshwater Ecology and Inland Fisheries (FV-IGB), Department of Ecohydrology, 12587 Berlin, Germany; z International Platform for Dryland Research and Education, Tottori University, Tottori 680-0001, Japan; aa Unité de Recherche en Science du Sol, INRAE, Orléans 45075, France; ab Sciences de la Terre et de l'Univers, Orléans University, 45067 Orléans, France; ac School of Biological Sciences, University of Adelaide, Adelaide, Australia; ad Faculty of Science and Technology, Free University of Bozen-Bolzano, Bolzano, Italy; ae Department of Watershed Management Engineering, Faculty of Natural Resources, Yazd university, Yazd, Iran; af Estación Experimental de Aula-Dei (EEAD-CSIC), Spanish National Research Council, Avenida Montañana, 1005, 50059 Zaragoza, Spain; ag State Key Laboratory of Earth Surface Processes and Resource Ecology, Faculty of Geographical Science, Beijing Normal University, Beijing, China; ah Institute of Land Surface System and Sustainable Development, Faculty of Geographical Science, Beijing Normal University, Beijing, China; ai University of Twente, Faculty of Geo-Information Science and Earth Observation (ITC), PO Box 217, Enschede AE 7500, the Netherlands; aj Team Soil, Water and Land Use, Wageningen Environmental Research, Wageningen 6708RC, Netherlands; ak Castilla La Mancha University, School of Advanced Agricultural and Forestry Engineering, Albacete 02071, Spain; al World Food Programme, Roma 00148, Italy; am University of Leicester, Centre for Landscape and Climate Research, Department of Geography, University Road, Leicester LE1 7RH, UK; an Julius Kühn Institute (JKI), Federal Research Centre for Cultivated Plants, Institute for Strategies and Technology Assessment, Kleinmachnow, Germany; ao Ludwig-Maximilian University, Munich, Germany; ap Southwest Watershed Research Center, USDA-ARS, 2000 E. Allen Rd., Tucson, AZ 85719, United States; aq Soil Research Institute, Council for Scientific and Industrial Research, Kwadaso, Kumasi, Ghana; ar Department of Agronomy and Horticulture, University of Nebraska-Lincoln, Lincoln, NE, United States; as Normandie Univ, Rouen, UNIROUEN, UNICAEN, CNRS, M2C, FED-SCALE, Rouen, France; at Romanian Academy, Iasi Branch, Geography Group, 8 Carol I, 700505, Iasi, Romania; au ISRIC - World Soil Information, Wageningen, the Netherlands; av Minas Gerais State University - Campus Frutal, Brazil; aw Georges Lemaître Centre for Earth and Climate Research - Earth and Life Institute, Université Catholique de Louvain, Belgium; ax Faculty of Agriculture, University of Torbat Heydarieh, Torbat Heydarieh, Iran; ay University Hassiba Benbouali of Chlef, Laboratory of Chemistry Vegetable-Water-Energy, Algeria; az Department of Physical Geography, University of Trier, 54296 Trier, Germany; ba Department of Veterinary Sciences, University of Pisa, Pisa, Italy; bb Faculty of Natural Resources, University of Tehran, Tehran, Iran; bc Department of Agricultural and Environmental Sciences, University of Milan, Via Celoria 2, 20133 Milan, Italy; bd Department of Chemistry and Biochemistry, University of Alaska Fairbanks, Fairbanks, AK, USA; be School of Environmental Ecology and Biological Engineering, Wuhan Institute of Technology, Wuhan 430205, China; bf Department of Plant Science and Landscape Architecture, University of Maryland, College Park, MD, USA; bg Department of Rural and Surveying Engineering, Aristotle University of Thessaloniki, 54124 Thessaloniki, Greece; bh Centre for Environmental and Marine Studies (CESAM), Dpt. of Environment and Planning, University of Aveiro, Portugal; bi Department “Agraria”, University “Mediterranea” of Reggio Calabria, Località Feo di Vito, 89122 Reggio Calabria, Italy; bj State Key Laboratory of Soil Erosion and Dryland Farming on the Loess Plateau, Institute of Soil and Water Conservation, Northwest A&F University, Yangling, Shaanxi 712100, China

**Keywords:** Erosion rates, Modelling, GIS, Land sustainability, Land degradation, Policy support

## Abstract

To gain a better understanding of the global application of soil erosion prediction models, we comprehensively reviewed relevant peer-reviewed research literature on soil-erosion modelling published between 1994 and 2017. We aimed to identify (i) the processes and models most frequently addressed in the literature, (ii) the regions within which models are primarily applied, (iii) the regions which remain unaddressed and why, and (iv) how frequently studies are conducted to validate/evaluate model outcomes relative to measured data. To perform this task, we combined the collective knowledge of 67 soil-erosion scientists from 25 countries. The resulting database, named ‘Global Applications of Soil Erosion Modelling Tracker (GASEMT)’, includes 3030 individual modelling records from 126 countries, encompassing all continents (except Antarctica). Out of the 8471 articles identified as potentially relevant, we reviewed 1697 appropriate articles and systematically evaluated and transferred 42 relevant attributes into the database. This GASEMT database provides comprehensive insights into the state-of-the-art of soil- erosion models and model applications worldwide. This database intends to support the upcoming country-based United Nations global soil-erosion assessment in addition to helping to inform soil erosion research priorities by building a foundation for future targeted, in-depth analyses. GASEMT is an open-source database available to the entire user-community to develop research, rectify errors, and make future expansions.

## Highlights

• Peer-reviewed research literature on soil-erosion modelling was reviewed.
• 66 soil-erosion scientists from 25 countries contributed to this study.
• Overall, 8471 articles identified as potentially relevant were reviewed.
• 1697 articles were reviewed in a comprehensive manner extracting 42 attributes.
• A free and open-source database was created.

## Graphical abstract

**Figure:**
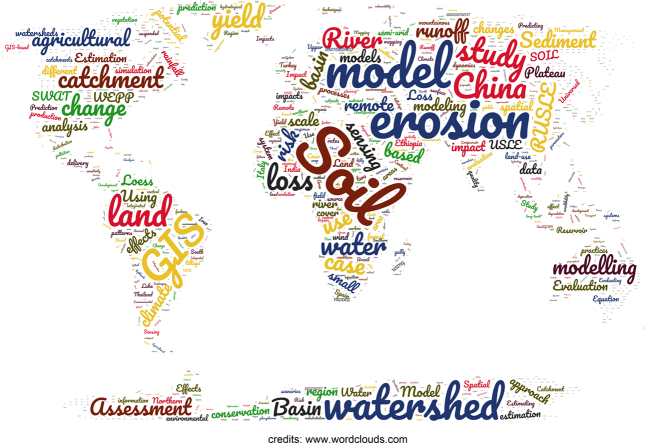

## 1 Introduction

Humans affect natural erosion processes and have induced a relevant and observable increase in soil erosion rates across landscapes (Poesen, 2018). For over a century the scientific community has been addressing the processes governing soil erosion, the occurrence of accelerated soil erosion, and its negative associated socio-environmental impacts (Bennett and Chapline, 1928; Smith, 1914). A body of research on the mechanics of soil erosion and its geographical distribution has benefited from the cognitive contributions of several adjoining disciplines, such as physical geography, soil science, engineering, hydrology, biogeochemistry, human sciences, and economics.This interdisciplinary nature is reflected in the numerous scientific approaches presented in the literature to better understand soil erosion phenomena, each having variable temporal and spatial scales, methodologies, and research goals (Boardman and Poesen, 2006; Morgan, 2009). Qualitative and quantitative descriptions of soil erosion have been performed through field observations and measurements (Toy et al., 2002), laboratory experiments (Mutchler et al., 2017), as well as through a meta-analysis of soil erosion rates across the world (García-Ruiz et al., 2015). Summatively, the vast and diversified scientific literature states that soil erosion includes a broad spectrum of processes (Poesen, 2018), which come with different characteristics (form, intensity, and frequency) and encompass all continents (Oldeman, 1994; Wuepper et al., 2020).

With an increased abundance of observed data and the aim of mapping spatially distributed soil erosion rates with a better understanding of their mechanics (Cook, 1937), scientists started to develop quantitative soil-erosion prediction equations based on physical factors such as climate, soil characteristics, vegetation type, and topography (Zingg, 1940). Since scientists proposed one of the earliest quantitative soil-erosion prediction equations in the 1940s, several mathematical models classified as empirical, conceptual, or process-oriented have been developed to predict soil erosion processes at different spatial and temporal scales (Merritt et al., 2003; Morgan and Nearing, 2011; Nearing, 2013). Batista et al. (2019) reported that today “there is no shortage of soil erosion models, model applications, and model users' but there is still a knowledge gap on the validity, quality, and reliability of the modelling application results”. Despite the significant progress made in model development and input parameterization, output uncertainties persist due to the non-linear relationships and thresholds at play between driving factors and the subsequent erosion processes, as well as the difficulties of upscaling model findings from the local scale to larger ones (De Vente and Poesen, 2005).

Part of the challenge to improve soil-erosion modelling is the development of baseline information on how models are used. Essential questions are: What do we know about soil-erosion model applications worldwide? What processes and models are mainly addressed? What are the regions where models are mainly applied? What are the regions that remain unaddressed? How frequently and how well are model outcomes validated? In short, we lack a clear picture of the worldwide state-of-the-art of soil-erosion model applications.

Today, with the well-established use of geospatial technologies like Geographic Information Systems (GIS), spatial interpolation techniques, and the ever-growing range of environmental data; soil-erosion models play an increasingly important role in the design and implementation of soil management and conservation strategies (Panagos et al., 2015b). The applications of soil erosion models are growing (Auerswald et al., 2014), alongside the scale of their application (Borrelli et al., 2017a, Borrelli et al., 2017b; Naipal et al., 2018). These models play an important role as tools to support decision-makers in policy evaluations (Olsson and Barbosa, 2019). The Sixth Session of the Global Soil Partnership (GSP) Plenary Assembly, under the solicitation of its Intergovernmental Technical Panel on Soils (ITPS), voted in favor of a resolution to put the development of a new country-driven global soil-erosion (GSER) assessment (GSP, 2019) on the agenda for 2019–2021. Unlike previous United Nations (UN) assessments that were based on expert judgments carried out in the 1990's, such as the Global Assessment of Human-induced Soil Degradation (GLASOD, Oldeman, 1994), the new UN Global Soil Erosion map (GSERmap) will rely on modelling. These modelling activities will be supported and validated by field and remote observations using satellite imagery and aerial photography. GSERmap will address the three main soil erosion-driven processes, i.e., water erosion, wind erosion, and redistribution due to the mechanization of agriculture (referred to as tillage erosion).

The new country-based UN global soil erosion assessment will involve hundreds of soil erosion experts worldwide (FAO, 2019). This represents an opportunity to enhance the understanding of global soil erosion, identify soil-erosion hotspots, and gain momentum for new policies at all levels. A UN project of this scale on soil erosion can also strengthen the soil-erosion scientific community's collaborative efforts to boost the development and applicability of models. However, the achievement of these goals could be hindered by the lack of global knowledge on soil erosion model usage. Improving such knowledge would help pave the way for more structured modelling and allow the further identification of needs to validate, measure, monitor, and map soil-erosion processes.

In this study, we systematically reviewed soil-erosion modelling applications worldwide and performed a statistical analysis with the aim of addressing identified knowledge gaps and facilitating information acquisition for the new country-based UN global soil erosion assessment. The subsequent database presents the current state of knowledge on soil-erosion modelling applications worldwide. We aimed to create and share a comprehensive and unprecedented database on soil erosion applications worldwide with an open science participatory approach. Sixty-six scientists from 25 countries representing all continents (except Antarctica) have contributed their findings, systematically reviewed all available peer-reviewed literature, and merged their knowledge. The database is available in Appendix A of this article. In our study, we provide an evaluation of (i) the processes and models most frequently addressed in the literature, (ii) the regions within which models are primarily applied, (iii) the regions which remain unaddressed and why and (iv) how frequently studies are conducted to validate/evaluate model outcomes relative to measured data. This approach provides insights into the worldwide state-of-the-art in soil-erosion model applications and allows a synthesis of information on which processes, models, and regions have received the most evaluative attention and which require increased focus in the future.

## 2 Methods

### 2.1 Data collection and GASEMT database

In this study, we report the results of an in-depth review of scientific peer-reviewed literature on soil-erosion modelling published in international journals between the 1st of January 1994 and the 31st of December 2017 and present in Elsevier's Scopus bibliographic database. We used the following criteria to identify articles potentially relevant for our statistical analysis: keywords *soil erosion* and *model* or *name of the model* (Box 1) in the title, abstract, or the keywords of the Scopus indexed articles. All articles matching the selected keywords have been downloaded and reviewed by one of the 67 soil erosion experts involved in the study. The review phase started in early 2018 and followed a participatory approach open to the entire scientific community, without any restrictions. The authors are composed of scientists who responded to an open call for expression of interest published on ResearchGate and advertised through mailing lists and word-of-mouth. Within the first data collection phase, all authors paid close attention to the following criteria: (i) verifying the relevance of the articles with respect to the objective of the review study, (ii) recording the entries' information (hereinafter also referred to as records), and (iii) extract all information listed in Table 1 for each relevant article. As a second quality control phase, P. Borrelli randomly inspected about 5% of the articles reviewed by the authors and verified whether the gathered information was complete. P. Borrelli reviewed the database to identify and rectify the most evident inconsistencies, misclassifications, and typos.

**Box 1:** Scopus query and acronym list of the soil erosion models used for the literature search (in the title, abstract, and the keywords of the Scopus indexed articles).

Scopus search
“soil erosion” AND “model” OR: AGNPS, ANSWERS, APSIM, CREAMS, EGEM, EPIC, EROSION-3D, EUROSEM, GeoWEPP, GLEAMS, GUEST, KINEROS, KINEROS2, LISEM, MIKE-11, MMF, MMMF, MOSES, MUSLE, PERFECT, PESERA, RHEM, RillGrow, RUSLE, RUSLE2, RUSLE-3D, RWEQ, SEDEM, SEDEM/WaTEM, SERAE, STREAM, SWAT, TMDL, USLE, USPED, WATEM, WATEM/SEDEM, WEPP, WEPS, WEQ.

**Table 1:** List of information collected for each entry in the GASEMT database (extended version in Table S1).

	Group	Entry	Types of data
i	Entry info	ID	Open (numeric)
		Reviewer ID	Open (alphanumeric)
		General ID	Open (alphanumeric)
ii	Bibliography	Year of publication	Open (numeric)
		List of authors	Open (alphanumeric)
		Title	Open (alphanumeric)
		Journal	Open (alphanumeric)
		DOI	Open (alphanumeric)
iii	Modelling exercise	Erosion agent	Multiple choice
		Modelling type	Multiple choice
		Gross/net estimate^a^	Multiple choice
		Quantitative/qualitative estimate^b^	Multiple choice
		Estimated soil erosion rate converted to (Mg ha^−1^ yr^−1^)	Open (numeric)
		Soil erosion rate (note)	Open (alphanumeric)
		Model name	Open (alphanumeric)
		Modelling aim	Multiple choice
		Modelled period	Multiple choice
iv	Study area	Continent	Multiple choice
		Country	Open (text)
		Name of the study area	Open (alphanumeric)
		Latitude (decimal degrees)	Open (numeric)
		Longitude (decimal degrees)	Open (numeric)
		Area (km^2^)	Open (numeric)
v	Climate	Data indicative period	Open (numeric)
		Type of data	Multiple choice
		Time resolution	Multiple choice
		Rainfall amount (mm)	Open (numeric)
		Rainfall (note)	Open (alphanumeric)
vi	Land use/cover	Type of data source	Multiple choice
		Modelled area	Multiple choice
vii	Fieldwork activities	Field activities	Multiple choice
		Type of activities	Multiple choice
viii	Soil info	Soil sampling	Multiple choice
		Type of soil information	Multiple choice
ix	Topography	DEM cell size (m)	Open (numeric)
x	Modelling outcomes	Scale^c^	Multiple choice
		Cell size (m)	Open (numeric)
		Modelled years	Open (numeric)
		Modelled period	Multiple choice
		Validation/evaluation attempt of model results	Multiple choice
		Type of validation/evaluation	Multiple choice
		Model calibration	Multiple choice

a Gross erosion is on-site soil erosion potential without considering re-deposition. Net erosion is the difference between erosion and deposition processes at a given point.

b Qualitative refers to an assessment of temporal trends, spatial patterns and/or driving factors, while quantitative refers to quantitative assessment of sediment detachment and or transport.

c Definitions are provided in the Supporting Information (Table S2).

The database is named Global Applications of Soil Erosion Modelling Tracker (GASEMT). In the case of studies reporting multiple model applications or numerous study sites, authors created multiple individual data entries in the GASEMT database. Each entry in the database reports information on the 42 attributes listed above (unless the reviewers did not find the required details and therefore reported the term ‘unknown’). The term ‘NA’ is used as the acronym of ‘not applicable’. Notably, the database only considers and reports on studies presenting soil-erosion modelling applications with spatially and temporally defined boundary conditions. In the database, we did not include data from articles that exclusively reported technical descriptions of models, refinement of individual model parameters, or methodological improvement without practical applications. We excluded all articles not written in English from the analysis.

### 2.2 Statistical analysis

GASEMT's records allow a comprehensive meta-analysis of soil-erosion model predictions, which cover a diverse range of time periods and locations globally. A subset of GASEMT data with complete inputs that included (i) modelled soil erosion rates (Mg ha^−1^ yr^−1^), (ii) geographical coordinates, and (iii) the size of the study area (km^2^), allowed statistical insights to be gained into soil-erosion model prediction patterns and trends through space and time. Excluding continental and global scale studies from this analysis, 1586 of GASEMT's modelling estimates met all these requirements, compiled from 786 individual publications.

Firstly, we approximated the global land surface (km^2^) covered by the recorded modelling applications together with data on total soil loss (billion Mg yr^−1^), the average (x̃) area-specific soil erosion (Mg ha^−1^ yr^−1^), and standard deviation (σ). Secondly, we analysed and subdivided modelling applications by categories of i) land cover/use, ii) type of erosion agent, iii) the scale of application (listed in Table 1). We used the non-parametric Kruskal–Wallis test to investigate the difference between the categories of records, accompanied by boxplots to display the distributions of predicted erosion rates among land use/land cover and different models (including minimum and maximum, first quartile, median, third quartile). Temporal trends were identified by means of simple linear regression.

## 3 Results

A literature search in the Elsevier's Scopus bibliographic database resulted in 8471 articles potentially reporting soil-erosion modelling applications. The further review process revealed that 6042 articles (71%) were not relevant for the study, as they did not report actual soil-erosion modelling applications. The number of articles not in English language or not accessible totalled 513 (6%) and 241 (3%), respectively. The resulting number of suitable articles was 1697 (20%), representing 3030 data entries in GASEMT, each equal to an individual modelling application.

### 3.1 Geography of the modelling applications

**Fig. 1:**
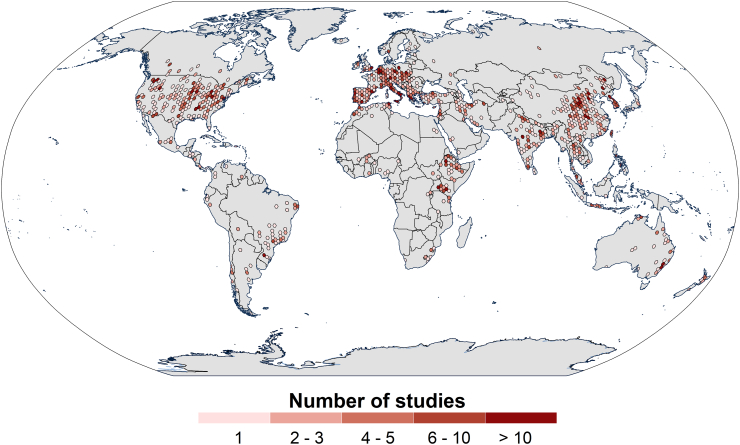
Geographical distribution of 1833 of the 3030 GASEMT database records for which the study areas' geographical coordinates could be obtained. The modelling applications are grouped using a hexagonal grid with a Robinson projection to represent the density of observations optimally.

Fig. 1 illustrates the geographical distribution of the modelling applications grouped using a hexagonal grid to optimally visualize the density of the observations. The 3030 individual modelling records are spread across 126 countries and all continents except Antarctica. These records cluster spatially into well-defined and identifiable geographical regions. Three areas of high application density could be observed around North America, Central/Southern Europe and Northeast/Far East Asia. In contrast, a lower application density can be observed in clusters covering the eastern sectors of South America, Africa and Oceania. Numerically, Asia (*n* = 976) and Europe (*n* = 929) show the highest number of modelling applications, followed by North America (*n* = 613) and to a lesser extent Africa (*n* = 251), South America (*n* = 123) and Oceania (*n* = 104). An inter-country analysis based on their number of records in GASEMT shows that the United States of America (537) and China (450) have the highest number of records in the GASEMT database, followed by India (161). Considering the European Union (EU-28, including the United Kingdom) as a single geographical entity, it would show the highest number of modelling applications, totalling 841 entries. In the EU, the highest frequencies are observed in Mediterranean countries such as Italy (*n* = 173), Spain (*n* = 125), and Greece (*n* = 84). In contrast, few model applications are available for large sectors of South America, Western and Central Africa, and North/Central Asia, with further decreased coverage in non-desert continental interiors. Overall, we noted a general tendency of the studies to be located within the main global cropland districts, as corroborated by further observations carried out and reported in the Discussion section.

### 3.2 Temporal trends

**Fig. 2:**
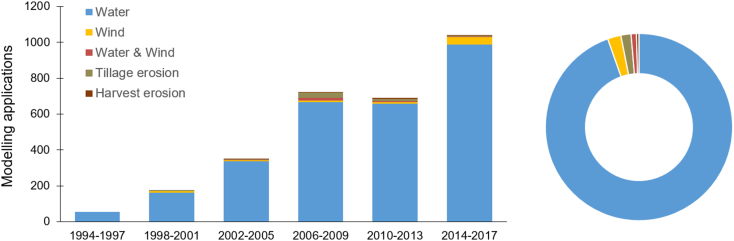
Number of publications catagorised by the simulated erosive agent in the GASEMT database through time (left panel, 4-year time windows) and overall 1994–2017 (right panel). Both panels share the same legend.

A total of 1697 articles applying soil-erosion models at local/regional/national or larger scales were published within the 24 years (1994–2017) covered by GASEMT, with an average publication rate of 70 articles per year. Splitting the database into 4-year time windows reveals an increasing trend of publications (Fig. 2), except for the 4-year period from 2010 to 2013. The last evaluated year (2017) recorded the highest number of annual publications (158 articles, 340 modelling applications). Studies on soil erosion by water dominate all 4-year time windows. In the first distinguished period (1994–1997), all 55 modelling applications reported in the database addressed soil erosion by water. During this period, 51 of these studies were performed within the three distinguished major spatial clusters i.e., USA (n = 17) and Canada (*n* = 6), India (*n* = 16), and European Union (n = 12). Models were mostly applied at watershed (*n* = 23) and plot scale (*n* = 19), with the median size of the investigated study areas being 0.43 km^2^. Interestingly, during the pioneering stage of the mid-nineties, soil erosion modelling did not lack large-scale applications (>1000 km^2^), e.g., Sharma and Singh (1995) in India and Pinheiro et al. (1995) in France. In this early period, Batjes (1996) published the first global assessment of land vulnerability to water erosion using a simplified version of the USLE model (Wischmeier and Smith, 1978). The most applied models during 1994–1997 belonged to the Universal Soil Loss Equation family (USLE/RUSLE; (Renard et al., 1997; Wischmeier and Smith, 1978), Productivity, Erosion and Runoff Functions, to Evaluate Conservation Techniques (PERFECT; Littleboy et al., 1989), Water Erosion Prediction Model (WEPP; Flanagan and Nearing, 1995), the Limburg Soil Erosion Model (LISEM; De Roo et al., 1996) and the Areal Nonpoint Source Watershed Environment Response Simulation (ANSWERS; Beasley et al., 1980).

In time windows post-1997, modelling applications investigating wind, tillage (downslope sediment redistribution due to tillage activity), and harvest erosion (export of sediments with harvested plants due to soil attaching to roots or machine parts) become apparent in the GASEMT database. In most time periods, the number of applications modelling this suite of processes are below ten. Only in 2006–2009 and 2014–2017 did tillage (*n* = 30) and wind erosion (*n* = 41) exceed ten database entries, respectively. Within 2014–2017, wind erosion models show an evident increase reaching a level of applications higher than observed in the previous 20 years. Most study areas with wind erosion modelling records are located in the USA (*n* = 32) and China (*n* = 18), while applications are mostly regional (*n* = 34) and plot scale (n = 30). Large scale modelling applications include five national (Baade and Rekolainen, 2006; Borrelli et al., 2015; Hansen, 2007; Hansen et al., 2002; Mezosi et al., 2015), two continental (Borrelli et al., 2016, Borrelli et al., 2017a, Borrelli et al., 2017b) and one global-scale application (Chappell and Webb, 2016). The most commonly applied wind erosion models are the Wind Erosion Equation ((R)WEQ; Fryrear et al., 2001; Woodruff and Armbrust, 1968), the Single-event Wind Erosion Evaluation Program (SWEEP; Wagner, 2013), and the Wind Erosion Prediction System (WEPS; Wagner, 1996).

### 3.3 Erosion processes and type of predictions

The GASEMT database has a marked dominance of water-erosion studies, constituting 94.6% of all entries. Roughly 0.9% of the data entries reported combined estimates of water and wind erosion, while individual simulations of wind (2.3%), tillage (1.8%), and harvest erosion (0.4%) also contributed small parts of the database (Fig. 2). The vast majority of the model applications estimate only sheet and rill erosion processes (~54%), with a smaller proportion estimating sediment yields (~27%) and sediment budgets (net erosion/deposition) (~10%). The remaining 10% of modelling applications can be classified as stream bank erosion (1%), mass movement (0.6%), rill (0.5%) and gully (0.3%), or more generally as sensitivity mapping (2.8%), soil displacement due to wind erosion (2.3%), and others (2.5%). Overall, the vast majority of modelling applications yield quantitative estimates of erosion (water erosion ~95%; wind erosion ~85%), whereas qualitative assessments represent ~5% of the entries. The term qualitative refers to an assessment of temporal trends, spatial patterns, or driving factors, while quantitative refers to a quantified assessment of sediment detachment and/or transport. Although around 95% of the entries report quantitative soil-erosion predictions, soil-erosion rates (in Mg ha^−1^ yr^−1^) could be retrieved only for one-third of the studies (*n* = 1890; 67% of the quantitative models). This is because the information was missing, not found by the reviewer, or illustrated in a figure.

### 3.4 Spatial scale

**Fig. 3:**
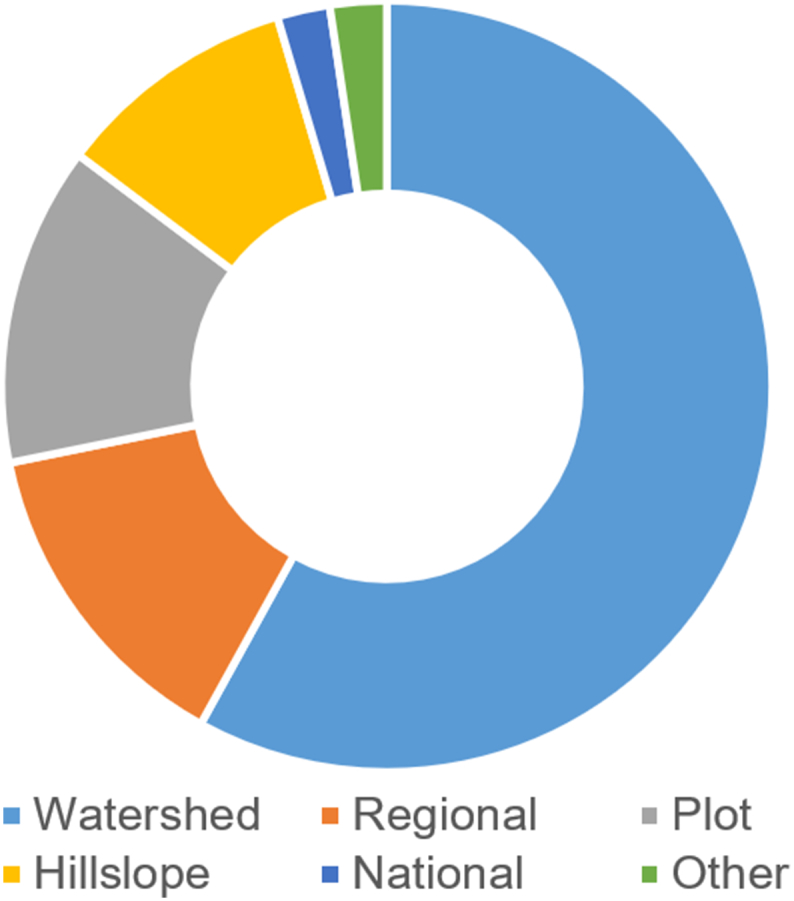
Distribution of the GASEMT database modelling applications according to spatial scale (other includes continental, farm, and global scale).

Global-scale soil erosion modelling applications represent ~0.6% (*n* = 20) of the total entries in GASEMT (Fig. 3). The vast majority of these global studies performed water-erosion estimations (n = 18) using (R)USLE family models (*n* = 17). Because the (R)USLE family is limited to sheet and rill processes, most of the global applications are limited to only these processes. The only non (R)USLE global water-erosion modelling application in GASEMT estimates the delivery of fluvial sediments to the coastal ocean through the BQART model (Syvitski and Milliman, 2014). The remaining two global modelling applications quantitatively estimate soil displacement due to water and tillage operations (Quinton et al., 2010) and the land vulnerability to wind erosion (Chappell and Webb, 2016).

Modelling applications at the continental scale represent 0.5% (*n* = 13) of the entries, eleven of which rest on quantitative estimates. Continental estimates have been made mostly in Europe (*n* = 11), followed by Africa (n = 1) and Oceania (n = 1) and the diversity of models applied is higher than at the global scale. In addition to classic models such as (R)USLE and (R)WEQ, eight other large-scale models estimating soil erosion at the continental scale have been applied (Borrelli et al., 2016, Borrelli et al., 2017a, Borrelli et al., 2017b; Bosco et al., 2015; Cerdan et al., 2010; Gericke, 2015; Hessel et al., 2014; Kirkby, 2006; Li et al., 2017; Van Oost et al., 2009; Panagos et al., 2015a; Podmanicky et al., 2011; Symeonakis and Drake, 2010; Teng et al., 2016). Eleven out of 13 modelling applications rested on quantitative estimation of soil erosion.

We identified 67 (~2%) national-scale modelling applications, mostly applied in Europe (*n* = 34), Asia (*n* = 12), and North America (*n* = 9). Except for three wind erosion studies in the USA and Spain (Baade and Rekolainen, 2006; Hansen et al., 2002), all other quantitative (*n* = 48) and qualitative (n = 3) applications focus on water erosion. Of these, the USA (*n* = 6), Czech Republic (n = 4), and Hungary (n = 4) are examples of countries with higher modelling applications.

About 14% (*n* = 418) of the recorded modelling exercises fall into regional-scale applications (x̃ = 6131 km^2^). Although smaller in size, the watershed-scale applications have the largest share of entries in the database (~59%), also including some very large study areas (x̃ = 128.5 km^2^). The three remaining small-scale application categories are hillslope (~10%; x̃ = 1 km^2^), farm/landscape (~0.4%; x̃ = 0.65 km^2^), and plot scale (~12.8%; x̃ = 0.0018 km^2^).

### 3.5 Aims of the modelling application

In ~40% of the GASEMT modelling applications, the authors did not describe their specific aim. In these cases, we classify the records as ‘general’ modelling exercises. They are to be considered as modelling applications carried out to generically assess the risk or magnitude of soil erosion without a specific aim. This contrasts to studies explicitly aiming to address land-use change, climate change, or their combined effects, which represent 20.4%, 3.5%, and 3% of the total, respectively. Other aims include the simulation of the impact of topographic change (3.7%), soil and water conservation (13.7%), ploughing impact (4.5%), forest harvesting (1.7%), wildfire (1.4%) and mining (0.3%). Studies simulating soil erosion dynamics in the present (52.4%), past (26.7%), or both (8.4%) represent most of the entries in the database (i.e., 87.5%). Although less common, studies providing either future or combined present and future projections of soil erosion still cover a relevant share of the entries with 3.8% and 5.9%, respectively. For the remaining entries (~2.8%), the modelling application's temporal frame was not specified (classified as ‘unknown’ in GASEMT).

More than half of the modelling applications estimate soil erosion considering all types of land uses/land covers present in the investigated area (*n* = 1575; ~54.4%). Agricultural areas in general, and exclusively arable land, are modelled specifically in only about 13.6% and 9.3% of the cases, respectively. The remaining modelling applications address forests (5.1%), grassland/rangeland (4.7%) and to a lesser extent bare soil (2.4%), pasturelands (1.4%), agroforestry (0.8%), riverbank (0.6%), and mine soil (0.1%). For the remaining ~7.5% of entries it was not possible to retrieve land-use/cover information.

Concerning the procedures employed to describe land-use/cover conditions, according to the studies that explicitly provided this information (~79% of the total), most of the studies used existing land-use maps (25.4%), created their maps through remote sensing (23.8%), or combined the two (12.3%). A considerable number of studies (18.1%), however, performed field mapping/observations. For the remaining ~20%, classification information was not available.

### 3.6 Models, input data and outcomes

Overall, 435 distinct models and model variants are listed in the GASEMT database, although several cases indicated that different nomenclature referred to the same modelling approaches. Table 2 lists the 25 most applied models and offers an example of the issue related to the heterogeneous model nomenclature (e.g., USLE, RUSLE and USLE-SDR, RUSLE-SDR, SEDD). In their different forms and applications, the models belonging to the (R)USLE-family are by far the most widely applied soil erosion prediction models globally, with over 1200 applications (~41% of the total). These numbers would be higher if USLE-type models such as WaTEM/SEDEM, EPIC, SWAT, and USPED were to be counted as members of the (R)USLE group. Modelling approaches independent from the USLE such as the process-based WEPP (*n* = 224; 7.4%), LISEM (*n* = 58; 1.9%), EROSION-3D (*n* = 30; 1%), the Pan European Soil Erosion Risk Assessment (PESERA, Kirkby et al., 2004) (*n* = 24; 0.8%), and the European Soil Erosion Model (EUROSEM, Morgan et al., 1998) (*n* = 17; 0.6%) together cover ~12% of total models. The next most common empirical models after (R)USLE are the Soil and Water Assessment Tool (SWAT, Arnold et al., 1998) (*n* = 183; 6%), the Water and Tillage Erosion Model, the Sediment Delivery Model (WaTEM/SEDEM, Van Oost et al., 2000) (*n* = 139; 4.6%), and the Morgan-Morgan–Finney ((R)MMF, Morgan et al., 1984) *n* = 61; 2%).

**Table 2:** Lists of the top 25 most applied soil erosion prediction models according to the records reported in the GASEMT database.

Model	Records	%	References
RUSLE	507	17.1	(Renard et al., 1997)
USLE	412	13.9	(Wischmeier and Smith, 1978)
WEPP	191	6.4	(Laflen et al., 1991)
SWAT	185	6.2	(Arnold et al., 2012)
WaTEM/SEDEM	139	4.7	(Van Oost et al., 2000)
RUSLE-SDR	115	3.9	–
USLE-SDR	64	2.2	–
LISEM	57	1.9	(De Roo et al., 1996)
Customized approach	53	1.8	–
MUSLE	52	1.7	(Williams and Berndt, 1977)
MMF	48	1.6	(Morgan et al., 1984)
AnnAGNPS	47	1.6	(Young et al., 1989)
RHEM	44	1.5	(Nearing et al., 2011)
Unknown	36	1.2	–
Erosion 3D	29	1.0	(Schmidt, 1991)
EPIC	25	0.8	(Williams et al., 1983)
PESERA	23	0.8	(Govers et al., 2003)
USPED	22	0.7	(Mitasova et al., 1996)
GeoWEPP	20	0.7	(Renschler, 2003)
RUSLE2	20	0.7	(Foster et al., 2001)
EPM	19	0.6	(Gavrilovic, 1962)
STREAM	19	0.6	(Cerdan et al., 2002)
RUSLE/SEDD	16	0.5	(Ferro and Porto, 2000)
DSESYM	15	0.5	(Yuan et al., 2015)
EUROSEM	15	0.5	(Morgan et al., 1998)

**Fig. 4:**
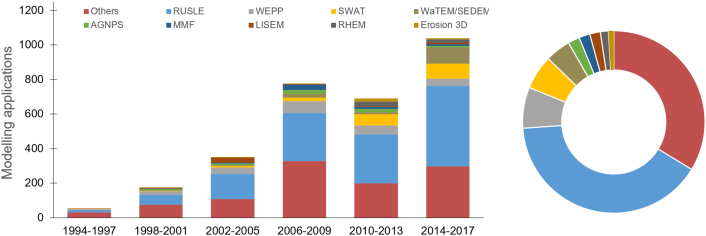
Number of publications according to models in the GASEMT database through time (left) (4-year time windows) and overall distribution (right).

The division into 4-year time windows (Fig. 4) indicates an evident increasing trend of (R)USLE, SWAT, and WaTEM/SEDEM usage, and to a lesser extent, WEPP, AGNPS, MMF, Erosion 3D, and LISEM. In contrast, the use of EUROSEM shows a negative trend over time.

Concerning model spatial resolution, surprisingly, such information was not reported in more than half of the modelling applications (~56%). From the reported studies, very high (≤ 5 m cell size) and high (> 5 m and ≤ 25 m cell size) spatial resolution modelling outputs respectively represent about 7.2% and 11.9% of the total. In most cases, these models are applied at the watershed, hillslope, and plot scales, although there are also a few national-scale applications (*n* = 10) and a pan-European one. Medium (> 25 m and ≤ 150 m) and moderate cell size (> 150 m and ≤ 300 m) outcomes were used for about 19.8% and 1.6% of the records, respectively. The remaining model applications (~3%) predicted soil-erosion rates with a coarse cell size between 330 and 60,000 m. Temporal analysis of the database shows a trend of decreasing cell sizes in modelled study areas at the watershed scale and below. Affinities between model type and grid-scale were not present except for in large-scale applications. These are mainly performed using empirical models of the (R)USLE and (R)WEQ families for water and wind erosion, respectively. Validation/evaluation of the modelling results was performed in most cases (~58%) in the 1697 artcles thoroughly reviewed in GASEMT. The most frequently used validation/evaluation method is the comparison of the modelling estimates against the measured sediment yield (SY) values (~26%). Comparisons against field-measured erosion rates, results of other models, and expert knowledge formed a total of ~18, ~10, and ~ 3%, respectively. Linear regression indicated that in the early period (1994–2000) of soil-erosion modelling the percentage of studies accompanied by validation/evaluation was higher. Although not statistically significant, we observed a slightly decreasing trend starting in 2015. The vast majority of non-traditional models – those only applied around one to five times – provide a validation/evaluation of the results. Of the most applied models, those with the highest share of validation/evaluation (>85%) are ANSWERS, PERFECT, USLE-M, DSESYM, and EUROSEM. SWAT and WaTEM/SEDEM both have values around 80%, while LISEM, WEPP, and MMF total 72, 66, and 63.3%, respectively. Applications of USLE and RUSLE models show reasonably high (63–69%) validation/evaluation values when applied to simulate SY. However, these values drop when validating/evaluating hillslope gross erosion estimates (RUSLE: 41%; USLE: 34%). Except for the modelling results validated/evaluated through measured SY and comparisons with results from other models, different forms of validation/evaluation are not adequately detailed in the current version of GASEMT. These were classified as ‘measured erosion rates’ or ‘expert knowledge’. These two categories are too broad and generic when considered a posteriori and should be better defined in future versions of the database. An extensive set of techniques are included in the validation/evaluation group, ranging from volumetric loss measurement (e.g., pins, cross-sections, contour gauge, and terrestrial laser scanning) to qualitative observations performed through field observations and remote sensing. About one-third of the entries reported model calibration. The models with the highest shares of calibration are SWAT, LISEM, WaTEM/SEDEM, and MMF. Specific information about the calibration techniques was not collected, as these were found to be highly variable and difficult to classify given the extensive range of models considered.

Some level of field-based data collection exists in over half of the modelling application cases. In-situ soil erosion measurements are the most common field activity associated with modelling, followed by field observation and soil sampling for modelling parametrization. Mapping of erosion features is relatively infrequent, totalling less than 3% of the field activities.

### 3.7 Statistical analysis

Overall, the model area covers an approximated total surface of 48.3 million km^2^. This area covers about 32% of the World's land area assuming (i) a total area of 149 million km^2^ and (ii) a marginal overlap between the modelled areas contributing to the GASEMT database. The predicted annual soil erosion totals 80.4 billion Mg yr^−1^, with an average area-specific soil erosion rate of 16.6 Mg ha^−1^ yr^−1^ (x̃ = 7.4 Mg ha^−1^ yr^−1^; σ = 39.8 Mg ha^−1^ yr^−1^). As expected, a significant difference between median values of gross (x̃ = 10 Mg ha^−1^ yr^−1^) and net (x̃ = 5.4 Mg ha^−1^ yr^−1^) erosion is observed. In the gross erosion category, all modelling applications that did not consider re-deposition are included (e.g., traditional (R)USLE-based models)). In contrast, the net erosion category includes modelling applications that predict sediment yield from a plot, hillslope, or watershed. Models spatially predicting explicit net soil erosion/deposition rates (named in GASEMT as sediment budget models, e.g., WaTEM/SEDEM) show a lower median value equal to 4 Mg ha^−1^ yr^−1^ (x̄ = 14.1 Mg ha^−1^ yr^−1^).

**Fig. 5:**
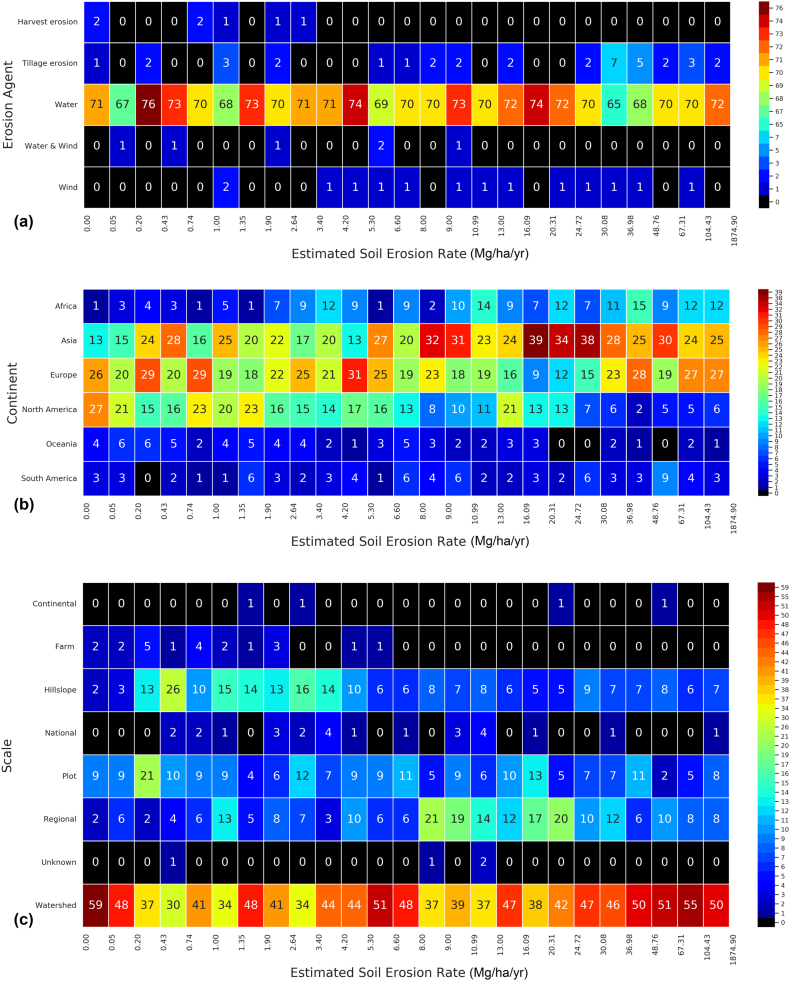
Distribution of the estimated soil-erosion rates (gross and net) categorized by erosion agent (panel a), continent (panel b), and spatial scale (panel c). Values in the cells and colour legend represent the numbers of occurrences in the database.

An analysis of estimated soil-erosion rates suggests that moderate to severe erosion is a common phenomenon under all climatic conditions encompassing all continents (except Antarctica). Fig. 5a shows that the vast majority of predicted soil-erosion rates refer to water erosion and to a much lesser extent to tillage (*n* = 37) and wind (*n* = 18) erosion. The predicted median values are 30.1 and 6.3 Mg ha^−1^ yr^−1^ for tillage and wind erosion, respectively. In terms of geographical region, the number of modelled occurrences of high and severe soil-erosion rates in Asia and Europe exceeds that in Africa, South America, and North America (Fig. 5b). Fig. 5c shows the categorical distribution of the predicted soil erosion rates scaled by intensity. Extremely high average rates (greater than 100 Mg ha^−1^ yr^−1^) of soil erosion are reported in 57 studies (76 entries), of which most are predicted in watershed-scale applications in Europe (~40%), Asia (~30%), and Africa (~17%). Surprisingly, most of the applications with extremely high erosion rates (73%) are so-called ‘generic modelling assessments’, which typically indicates that a model has been applied to heterogeneous land cover/use that includes natural and semi-natural vegetation (e.g., unmanaged grassland, bushland). Therefore, these studies did not target specific land disturbances such as wildfires, forest logging, or land-use changes for which severe soil erosion can be associated. Approximately 18% of the modelling applications reported in the GASEMT database aimed at land use or climate changes as the modelling objective.

**Fig. 6:**
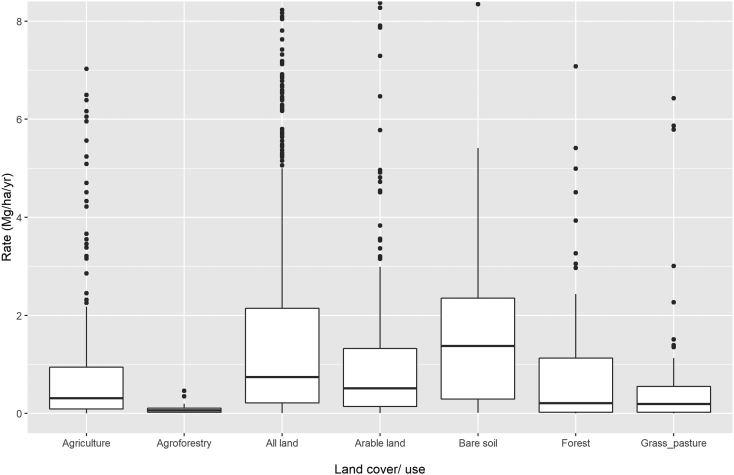
Comparison of modelled erosion rates under different land covers. Note that the outliers >8 mm yr^−1^ are excluded in the graphic. The boxplots display the interquartile range (grey boxes), the median (horizontal bold black lines), the 10th and 90th percentile (horizontal black lines) and outliers (dots).

Comparing soil-erosion rates by land cover/use types (Fig. 6), we observe a substantial decline in soil-erosion rates (reported in mm yr^−1^ assuming an average bulk density of 1.35 g cm^−3^) from bare soil (x̃ = 1.2 mm yr^−1^) to agricultural areas (generic, x̃ = 0.3 mm yr^−1^; arable land, x̃ = 0.5 mm yr^−1^; agroforestry, x̃ = 0.1 mm yr^−1^), forests (x̃ = 0.2 mm yr^−1^) and other forms of semi-natural vegetation (x̃ = 0.2 mm yr^−1^). When all land uses are modelled, we obtain a median value of 0.75 mm yr^−1^. This distribution of soil erosion rates among the different land cover/use units fit those reported by Montgomery (2007) and Borrelli et al., 2017a, Borrelli et al., 2017b for values observed from field measurements. However, the agreement is better for the values predicted in agricultural areas than those predicted in the grass and forestland areas.

The non-parametric Kruskal-Wallis test confirmed the absence of a statistically significant difference between measured soil erosion rates and those measured in arable lands. Modelled grass and forestland rates show a tendency to exceed their field measurement counterparts with median values in the order of 0.2 mm yr^−1^, considerably higher than those observed in field measurements which are placed at 0.001 and 0.01 mm yr^−1^ for forest and semi-natural vegetation, respectively. The disagreement between modelling results and field measurements could be partially explained by the fact that in more than 50% of the modelling exercises considering forestland and grassland areas changes in land cover/use or vegetation disturbances are reported. Cerdan et al. (2010) hypothesized that field measurements in arable lands could be biased towards areas known to be exposed to erosion processes. Similarly, our analysis results lead us to hypothesize that the modelling applications explicitly addressing forestland and grasslands could be biased towards areas experiencing human-induced disturbances.

**Fig. 7:**
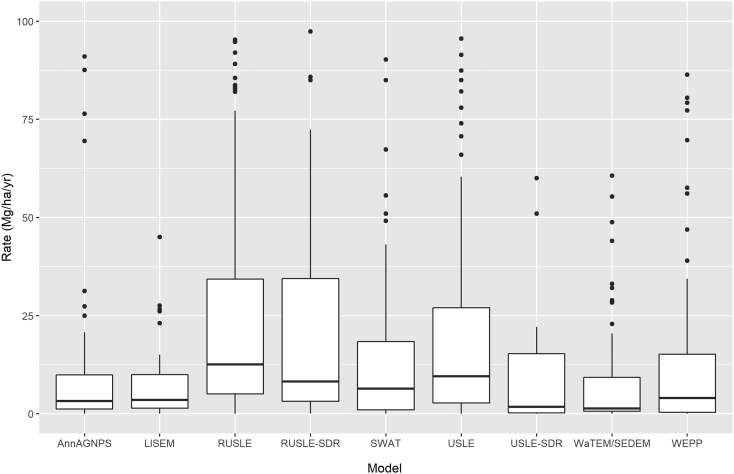
Comparison of the predicted soil erosion rates of the nine models most commonly occurring in the GASEMT database. Note that the outliers >100 Mg ha^−1^ yr^−1^ are excluded in the graphic. The boxplots display the interquartile range (grey boxes), the median (horizontal bold black lines), the 10th and 90th percentile (horizontal black bars), and outliers (dots).

The boxplots in Fig. 7 illustrate the key descriptive statistics of the soil-erosion estimates derived from the nine most commonly encountered models in the GASEMT database. Soil erosion rates predicted by models classified within the ‘net erosion’ group (and thus evaluating the budget between soil erosion and deposition either on plot scale or as net sediment transfer to downslope locations), such as AnnAGNPS (x̃ = 3.3 Mg ha^−1^ yr^−1^), LISEM (x̃ = 3.5 Mg ha^−1^ yr^−1^), SWAT (x̃ = 6.4 Mg ha^−1^ yr^−1^), WaTEM/SEDEM (x̃ = 1.4 Mg ha^−1^ yr^−1^), and WEPP (x̃ = 4.0 Mg ha^−1^ yr^−1^), show both a lower spread and median values compared to the models classified within ‘gross erosion group’, i.e., RUSLE (x̃ = 12.6 Mg ha^−1^ yr^−1^) and USLE (x̃ = 9.6 Mg ha^−1^ yr^−1^). In GASEMT, USLE-type models adopting a sediment delivery ratio (SDR) to estimate sediment yields are classified as RUSLE-SDR (x̃ = 8.3 Mg ha^−1^ yr^−1^) and USLE-SDR (x̃ = 1.8 Mg ha^−1^ yr^−1^). Models predicting net erosion (sediment yield) show average values lower than those simulating only gross erosion (RUSLE and USLE). This condition is more evident for the USLE-SDR models than the RUSLE-SDR models. A further observation of the boxplots shows that, except for the most commonly applied RUSLE model (345 records or 32% of the total), all other models have a median value below the 10 Mg ha^−1^ yr^−1^. Overall, models simulating gross erosion rates show higher values with higher variability than models predicting net erosion, reflecting (i) sediment deposition within the landscape, and (ii) the smoothing of extreme values by incorporating topographic variability in the net erosion models.

**Fig. 8:**
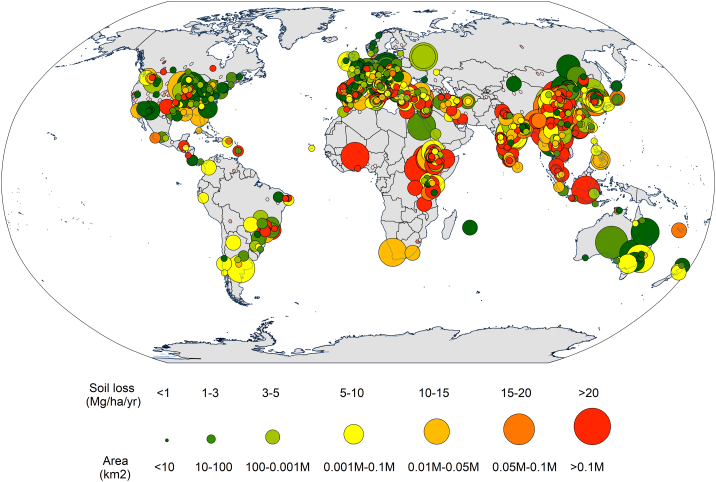
Geographical distribution of the 1586 quantitative modelling estimates, including the study area's size (proportional to the size of circles) and predicted soil erosion rates (chromatic scale). Robinson projection.

Fig. 8 shows the geographical distribution of the modelling estimates from the subset of 1586 studies. The circle sizes are proportional to the size of the study area, while the chromatic scale symbolizes the magnitude of the predicted erosion rates. As illustrated, quantitative estimates of soil erosion are available in all continents (except Antarctica) and under all climatic conditions, although the distribution is highly non-uniform. Aggregating estimates per general climatic zone reveals evident latitudinal trends, with the highest average values in the tropical zones (x̄ = 29.1; x̃ = 11.2; σ = 51.3 Mg ha^−1^ yr^−1^; 20.5% of the sites), steadily decreasing through subtropical zones (x̄ = 29.5; x̃ = 9.1; σ = 102.2 Mg ha^−1^ yr^−1^; 34.4% of the sites), temperate zones (x̄ = 16.1; x̃ = 4.1; σ = 33.7 Mg ha^−1^ yr^−1^; 44.2% of the sites), and polar and subpolar zones (x̄ = 3.0; x̃ = 1.4; σ = 3.7 Mg ha^−1^ yr^−1^; 0.9% of the sites). High predicted values (x̃ > 20 Mg ha^−1^ yr^−1^) could mainly be observed in Africa (Rwanda, Mauritius, Burkina Faso, Ghana, Kenya, Congo, Malawi, and Somalia), and to a lesser extent in Asia (Lebanon, Tibet, and Jordan), Europe (Portugal, Italy, and Greece), Southeast Asia (Malaysia and Indonesia), and South America (Nicaragua).

## 4 Discussion

The collaboration of 67 scientists from 25 countries representing all continents (except Antarctica) allowed the creation of GASEMT. The database is composed of 3030 individual modelling records (applied in 126 countries), retrieved from 1697 articles that were thoroughly reviewed. The database contains information on most of the existing peer-reviewed literature reporting spatially-explicit soil- erosion modelling applications. Accordingly, studies reporting only theoretical descriptions of models or enhancements of single models, components, or parameters were not considered suitable for the analysis. Similarly, studies reporting on measurements only, e.g., the analysis of fallout radionuclides as indicators of erosion processes (Lizaga et al., 2018; Mabit et al., 2019), were not taken into account.

With a set of 42 different attributes retrieved from each reviewed article (depending on availability), GASEMT constitutes a source of pre-structured literature information and references. The large number of records of this database makes it a highly practicalsource of information and a powerful tool for further research. Here, our attention and interest mainly address the observation and description of the general aspects of the soil-erosion modelling applications worldwide. However, we believe that GASEMT can be useful to comprehensively target a number of further in-depth studies and observations. Further analysis could disaggregate the information reported in GASEMT to address specific erosion agents and processes, methodologies, or geographical regions. We provided all details in the Supplementary Information (Appendix A) of this study. Bezak et al. (2021) provide a practical example of the GaSEM database's further use, investigating the relationship between soil erosion modelling and bibliometric characteristics (applying a generalized boosted regression tree model).

In the following, we discuss the implications of our results linking the findings obtained by the analysis of GASEMT to (i) evaluate which processes and models are primarily addressed in the literature, (ii) in which regions models are mainly applied, (iii) what regions remain unaddressed and (iv) how frequently validation/evaluation attempts of the model outcomes were performed with measured data.

*Evaluation of the processes and models primarily addressed in the literature*. In a recent review study, Poesen (2018) addressed the need for more research in understanding both natural and anthropogenic soil erosion processes. Borrelli et al., 2017a, Borrelli et al., 2017b noted a disparity in the literature between wind and water erosion studies in Europe, in terms of knowledge depth, number of peer-reviewed publications, and the number of ongoing field experiments. Today, a search in Scopus using the terms ‘erosion and water’ results in 52,730 mentions in publications, ‘erosion and wind’ is found in ca. 9488, ‘erosion and gully’ in ca. 3896 publications. In contrast, ‘erosion and piping’ and ‘erosion and harvest’ are found only in 1556 and 1037 documents, respectively (Scopus, 21.02.2020). These numbers provide a primary indication that over the last decades more attention has been dedicated to water erosion, therefore presumably more research, process description, and understanding. In contrast, other erosion processes seem to remain local environmental threats and thus have attracted less interest (Bernatek-Jakiel and Poesen, 2018; Panagos et al., 2019; Poesen, 2018; Van Oost et al., 2004). Information on spatial modelling applications reported in GASEMT confirms a lack of variety in the soil-erosion processes addressed. Notably, around 95% of modelling applications predicted water as the erosional agent, in contrast to few applications dealing with wind (39), tillage (23), or harvest erosion (3) processes. This means that ~85% of models and their varients developed so far have addressed water erosion, and in particular the vast majority (estimated between 50 and 80%) of these have focussed on the prediction of sheet and rill processes. We argue that this disproportionate attention dedicated to sheet and rill processes as erosion agents may poorly reflect their importance in terms of spatial extent and magnitude (Boardman and Poesen, 2006; Lal, 2007; Oldeman, 1994). Instead, the marked focus on sheet and rill erosion may be due to i) the current state-of-the-art in process understanding, ii) their established applicability to agricultural decision making, iii) the availability of measurement and modelling tools, and iiii) their successful coupling with GIS interfaces. In addition, the lack of literature on soil-erosion modelling from large regions where wind erosion is widespread, such as Asia (e.g., Russia), may contribute to this inequality.

Water-erosion models have been increasingly coupled with GIS interfaces during the last few decades, thus allowing the upscaling of soil erosion assessments from field to watershed scale and above. Upscaling has helped focus land-management decisions, e.g., allowing for greater precision in identification of higher erosion risk areas. At the same time, quantitative attempts to integrate wind and tillage erosion prediction models into GIS environments have been less straightforward, although quite a few applications have reached beyond the field scale. For instance, the latest reference document of the UN (FAO and ITPS, 2015) reports that a likely range of global soil erosion by water is 20–30 Gt yr^−1^, while tillage erosion may amount to ca. 5 Gt yr^−1^. These numbers, presumably based on the study of Quinton et al. (2010) reported in GASEMT, suggest that tillage erosion could account for up to 25% of water erosion globally. Modelling results reported by Borrelli et al., 2017a, Borrelli et al., 2017b indicated that wind erosion might be a relevant phenomenon for Europe, although this land-degradation process has been overlooked until very recently. Their estimates suggest soil erosion values can be particularly high for the arable land of Denmark (~3 Mg ha^−1^ yr^−1^), the Netherlands (2.6 Mg ha^−1^ yr^−1^), and the United Kingdom (~1 Mg ha^−1^ yr^−1^); indicating that wind erosion may be a major agent of soil erosion in localised areas. Quantitative assessments of wind erosion over large areas in China and Iran reported in GASEMT show average soil-erosion values well above 10 Mg ha^−1^ yr^−1^ (Jabbar et al., 2006; Rezaei et al., 2016; Zhang and McBean, 2016). In addition, the 37% of the GASEMT records reporting wind and tillage erosion predicted high soil erosion rates (x̃ = 10.2 Mg ha^−1^ yr^−1^) that may locally represent a threat to agricultural productivity and the sustainability of the Earth's natural resources.

Broad spatiotemporal trends in model applications are evident globally. In their different forms and applications, models belonging to the (R)USLE-family are by far the most widely applied soil-erosion models globally. They cover ~41% of the total records in the database. This value could increase to ~55% if USLE-based models such as WaTEM/SEDEM, EPIC, SWAT were included in the same category. In line with the observation of Alewell et al. (2019), we also found a strong rising trend (R^2^ 0.82 significance level < 0.001) of (R)USLE-type applications across all continents. Other models showing both rising trends and worldwide applications are SWAT (R^2^ 0.78 significance level < 0.001), WEPP-type (R^2^ 0.27 significance level < 0.01), WaTEM/SEDEM (R^2^ 0.27 significance level < 0.01), and to a lesser extent RHEM (R^2^ 0.21 significance level < 0.02) which remains almost exclusively applied in the United States of America. Other models had either no significant trend (MMF-type, LISEM) or had slightly negative trends (EUROSEM). In 2017, the last year of our observations, RUSLE-type applications (*n* = 153) were used many times more frequently as the most commonly applied process-based models, i.e., WEPP (*n* = 11), RHEM (*n* = 6), PESERA (*n* = 2), LISEM (n = 1), EUROSEM (*n* = 0).

*Regions where models are mainly applied*. We analysed the spatial distribution of modelling applications using a subset of 1833 records for which the spatial coordinates of their centroids could be gathered (shown in Fig. 1). The worldwide increase in usage of models with low input demand, such as (R)USLE-type, SWAT, and WaTEM/SEDEM, is accompanied by a significant rise in the size of the modelled areas (R^2^ 0.41 significance level < 0.001). The geographical distribution of soil-erosion modelling itself is clustered within well-defined geographical regions in North America, Europe and Southeast Asia. We found six countries to possess about 50% of the total modelling studies (i.e., United States of America, China, Italy, India, Spain and Australia). A higher incidence of modelled sites in temperate and subtropical zones can be also observed, while the occurrence in tropical regions is notably lower (~15%). This situation contrasts with a general understanding of the geography of soil-erosion processes emerging from field observations, indicating that tropical zones are more prone to erosion (Boardman, 2006). A phenomenon also confirmed by global expert-based qualitative assessments such as (GLASOD; Oldeman, 1994) and quantitative modelling descriptions of major soil-erosion drivers (Chappell and Webb, 2016; Panagos et al., 2017), which indicate tropical regions as being highly susceptible to erosion (Labrière et al., 2015). The GASEMT database demonstrates the lower incidence of studies in the tropics and subtropical regions, while latitudinal trends indicate higher soil erosion on average in the tropics (Fig. 8). The gradually increasing erosion rates from the subpolar zones to the temperate, the subtropical, and finally the tropics are paired with decreasing investigation intensity and thus a noticeable lack of knowledge. This situation indicates that the urgency of environmental impact assessment does not necessarily drive erosion modelling, but more the spatial occurrence and frequency of studies in the countries publishing the most science articles in peer-reviewed journals (Forbes, 2020). García-Ruiz et al. (2015) observed in a similar study evaluating measured soil erosion rates that their spatial occurrence does not necessarily reflect the regional relevance of soil erosion processes, but rather the spatial concomitance of soil-erosion processes with scientific groups interested in this topic and publishing their research outcomes in international literature. The overall volume of research on soil erosion modelling may be considerably larger, as suggested by the 419,000 results obtained searching for ‘*soil erosion modelling’* in Google Scholar.

**Fig. 9:**
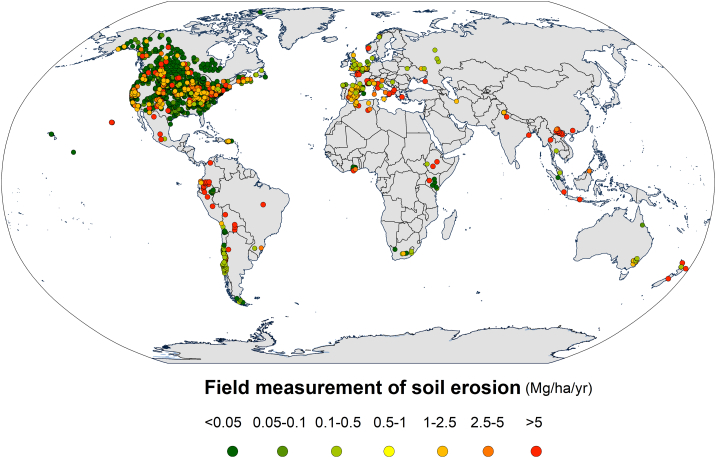
Spatial distribution (Robinson projection) of the sites reported in García-Ruiz et al. (2015) database on soil erosion field measurements.

A comparison of the spatial patterns of the soil erosion rate measurements collected by García-Ruiz et al. (2015) (Fig. 9) and the modelling applications gathered in this study (Fig. 8) indicates that model applications have a more even distribution globally. Although a significant spatial agreement between the two datasets can be observed in North and South America, Western Europe, and Eastern Africa, models appear to be more applied in regions that rarely report field measurements such as India, China, and Southeast Asia. While it is generally agreed in the scientific world that models should be validated/evaluated with measured data, erosion measurements are often as uncertain as modelling (Batista et al., 2019; Alewell et al., 2019), and are not in existence in many areas of the world. As such, modelling endeavours must be seen as hypotheses on temporal trends, spatial patterns, driving factors, and triggering processes.

*Regions unaddressed by modelling*. Without considering global and continental-scale studies, plot- to national-scale modelling applications would jointly cover a surface of approximately 48 million km^2^, equal to 32% of the world's land. This estimate assumes marginal overlap between the modelled areas within the GASEMT records. Further analysis/refinement of the data, excluding the most apparent spatial overlaps, results in about 35 million km^2^ of modelled land, or a realistic range between 25 and 35 million km^2^. Of this 35 million km^2^, about 66% is due to national-scale studies in the USA (~28.4%), China (~27.7%), and India (~9.5%). As expected from model application frequency, soil erosion by water dominated most of the modelled area, leaving wind and tillage erosion with values at approximately 2.5 and 0.12 million km^2^, respectively.

**Fig. 10:**
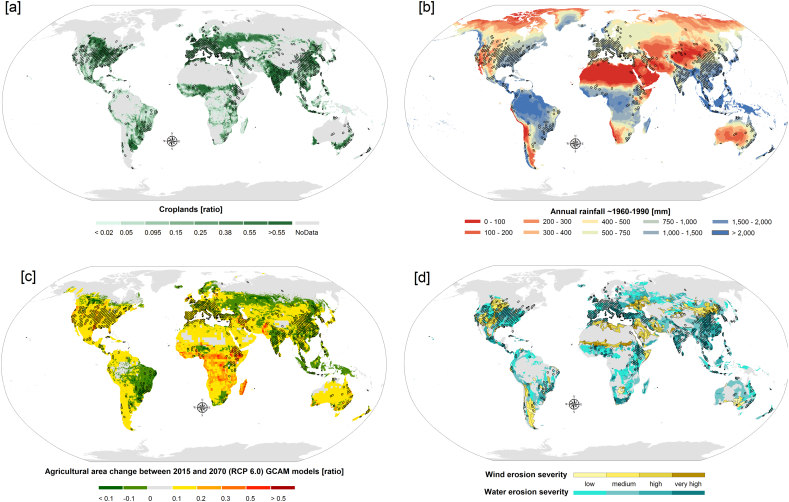
Geographical distribution (Robinson projection) of 1833 Global Applications of Soil Erosion Modelling Tracker (GASEMT), grouped using a hexagonal grid, superimposed on (panel a) the global cropland according to the IMAGE model year 2015 (Hurtt et al., 2020; Stehfest et al., 2014), (panel b) global annual rainfall (Hijmans et al., 2005), (panel c) global yearly changes in the agricultural area between the reference period 2015 and 2070 projections (Global Change Assessment Model (GCAM) RCP 6.0, Hurtt et al., 2020), and the water and wind erosion severity according to the Global Assessment of Soil Degradation (GLASOD) (panel d). The degree of damage is indicated from low (1) to severe (4). This figure is available at high-resolution in the Supplementary Information (Fig. S3).

We noted a general tendency of studies to be located around the main global cropland areas. These insights are corroborated by Fig. 10a, which overlaps the hexagonal pixels of modelled areas to global croplands (Hurtt et al., 2020; Stehfest et al., 2014). Based on the available peer-reviewed English-language journals, large areas exploited for crop production in Russia and East Europe, Central Asia, throughout most of Africa, and South America seem to be poorly studied through soil-erosion modelling. However, this can also be the result of more publishing in the local language or technical reports.

Fig. 10b presents the average annual rainfall for the period 1960–1990 (www.worldclim.org). Comparing rainfall patterns in Fig. 10b with the soil-erosion modelling applications indicates that areas characterized by low to medium rainfall values have been more intensely studied compared to regions in wet climates covered or formerly covered by tropical rainforest. These conditions are particularly noticeable along areas characterized by high rainfall erosivity in South-Eastern Asia (Cambodia, Indonesia, Malaysia, the Philippines, and Bangladesh), Central Africa (Congo and Cameroon), South America (Brazil, Colombia, and Peru), Central America, and the Caribbean (Panagos et al., 2017). Some of the regions poorly represented by soil-erosion modelling studies have experienced, and will probably continue to experience (Global Change Assessment Model (GCAM) RCP 6.0, Hurtt et al., 2020) (Fig. 10c), increasing trends of forest logging and cropland expansion (Hansen et al., 2013). This vulnerability could also be accompanied by a trend towards increasing rainfall intensities in these regions, as predicted by several future projections (Hijmans et al., 2005). Modelling projections suggest that tropical countries such as Peru, Brazil, several countries in Western Africa, Cameroon, Ethiopia, Somalia, Kenya, Yemen, Southern Pakistan, India, Myanmar, Southeast China, Philippines, and Indonesia may be significantly affected by increased soil erosion (Borrelli et al., 2020). In addition, roughly two and a half billion people currently live in these countries and their populations show signs of significant expansion (Bongaarts, 2016). Fig. 10d shows a strong spatial agreement between soil-erosion modelling study areas and the areas reported to be affected by water and wind erosion by the expert-based GLASOD, promoted by United Nations Environmental Programme. GLASOD was developed from the combination of data provided by more than 300 scientists from several countries. Although qualitative in nature, and potentially affected by the different perceptions of the contributing scientists, the GLASOD is still based on extensive field observations. The comparison between the patterns of the application of soil-erosion modelling and the independent GLASOD map provides insights into where modelling is being applied compared to its perceived needs. It should be noted that GLASOD refers to observations made during the 1980s and important global land-use changes have occurred in the following decades, which GLASOD does not reflect.

*Frequency of model validation/evaluation attempts of modelling outcomes against measured data*. Validation/evaluation of modelling results was applied in most of the cases in GASEMT (~58%). Plot-scale modelling (13% of the total records) shows higher levels of validation/evaluation (~68%) and calibration (~38%), mostly performed through volumetric measured erosion rates (37.8%) and collecting sediment yield (25%). Rising trends in studies reporting validation/evaluation (R^2^ 0.77 significance level < 0.001) and calibration (R^2^ 0.92 significance level < 0.001) procedures could also be observed, with recent higher values related to sediment-delivery models such as SWAT, WaTEM/SEDEM, and LISEM. However, as a proportion of total annual modelling applications, the trend in the number of models validated/evaluated and calibrated is negative. For example, a transition is observable from 80 to 90% of studies validated/evaluated in 1995–2000 to 60–70% in 2015–2017. The aforementioned trend might partly be due to the recent increase in modelling studies from countries and regions with a low measurement density (Fig. 8, Fig. 9).

Validation/evaluation procedures based on the comparison of observed versus simulated sediment loads at the outlet cover the largest record share (*n* = 798 GASEMT records). While the application of this type of validation/evaluation seems most logical for prediction at plot scale (*n* = 102) (Nearing, 2000), outlet-based validations/evaluations applied at the watershed scale (*n* = 605) leaves room for concern regarding the effectiveness of the validation/evaluation procedure (Borrelli et al., 2014; De Vente and Poesen, 2005). As derived from our analysis, most of the models validated/evaluated through outlet-based procedures (SWAT, WaTEM/SEDEM, and (R)USLE-SDR, among others) provide estimates of soil erosion only due to sheet and rill processes. Most models cannot directly or indirectly account for the soil displacement due to gully and tillage erosion processes. Most do not model other geomorphic processes such as landslides, riverbank erosion, and riverbed and floodplain deposition. Other validation/evaluation methods more commonly encountered in GASEMT records are based on measured erosion rates (*n* = 536). Unexpectedly, a substantial proportion evaluated model performances come through comparisons with other model simulations (*n* = 305).

Published studies profusely described the lack of data and knowledge which currently limits the capacity to perform validations sensu *strictu* (Auerswald et al., 2003) of modelling results beyond plot and hillslope scales (Alewell et al., 2019; Auerswald et al., 2003; De Vente et al., 2013; De Vente and Poesen, 2005). Knowledge gained from our analysis indicates that many soil-erosion models are applied deterministically (without validation/evaluation of the results). However, both small- (plot and hillslope) and large-scale applications (watershed and regional) show rather high levels of validation/evaluation attempts (understood here as a comparison of modelled to observed data or modelled to a second independent modelled data set) of the results (above 45% in all cases). Nevertheless, while the comparisons versus in situ measured erosion rates result in appropriate validation/evaluation procedures for plot and hillslope scale predictions, the lack of long-term observations and measurements at the watershed to regional scale poses serious limits to models applied at such scales.

As pointed out by this review study, a large number of models have been developed over the last three decades. They range from simplistic expressions to highly articulated and complex models able to consider a large number of interacting factors and physical relationships. Models listed in GASEMT can be classified into three modelling categories: empirical, semi-empirical, and physically-based (or process-oriented). According to GASEMT records (Table 2), the ten most applied soil erosion models include all these three categories. USLE-type models dominate both, empirical (USLE, RUSLE, and AnnAGNPS) and semi-empirical (SWAT, WaTEM/SEDEM, MUSLE) models. The remaining four models are either semi-physically based models, like MMF, which has characteristics of both physically based and empirical models, or physically-based ones such as WEPP, LISEM, and RHEM.

The three most applied empirical models are characterized by different evolutional stages of USLE equations. USLE had its roots in the 1930s when the US government passed the Soil Conservation Act. During the same period, President Franklin D. Roosevelt stated: “The history of every Nation is eventually written in the way in which it cares for its soil.” USLE is a result of statistical analysis of more than 10,000 plot-years of basic runoff and soil loss, using plots with less than or equal to 122 m and a slope of 3 to 18%. The relationship between soil loss, rainfall erosivity and soil type is corrected using information on slope steepness, slope length, crop cover, and anti-erosive measures management. As with all empirical methods, Alewell et al. (2019) observed that the model concept is not based on process descriptions and simulations. Rather it rests on understanding the processes, capturing the confounding *measureable* parameters, and delineating a mathematical algorithm from the relationship between these parameters and the measured output, i.e., measured eroded sediments. Based on statistical relationships, the model's empirical nature influences the required computational processes, which makes it relatively simple and keeps data requirements affordable. USLE-type models predict long-term annual averages of soil loss. They belong to the detachment limited model type. This means that although the overland flow may theoretically transport an infinite sediment amount, the quantity of sediment available to be moved is limited by the soil detachment capacity defined by the rainfall's erosivity. Annual soil loss per unit area and time (Mg ha^−1^ yr^−1^) is given by a multiplicative equation of six factors: driving force (erosivity of the climate, R), a resistance term (erodibility of the soil, K), as well as other factors representing farming choices, i.e., the topographical conformation of the field (LS), cropping system (C) and soil conservation practices (P). A set of six primary factors and less than 20 sub-factors are generally needed to predict soil loss in a given physiographic unit (slope, watershed, and region). The main limitation of empirical models for predicting soil erosion is their inability to be applied outside the geographical conditions where their statistical relationships were derived from. Despite plot measurements of 49 US locations in a large variety of landscape conditions, Wischmeier and Smith (1978) noted that insufficient measured data exist to rigorously determine the single factors for all needed situations and scenarios. Nearing (2004) observed that USLE-type models also have limitations in their structure, allowing only for limited interactions and inter-relationships between its basic multiplicative factors. Considering current modelling applications and needs, another major limitation of USLE-type models is the absence of algorithms predicting deposition and sediment yields.

Semi-empirical models (i.e., MUSLE, WaTEM/SEDEM, SWAT) listed in GASEMT are a combination of empirical and physically-based models, where the empirical component is the dominant component. The major advance compared to the traditional USLE applications is the integration of equations to describe (simplistically) erosion and sediment transport processes in order to predict sediment yields. MUSLE and SWAT share the same modelling scheme with a minimal difference. They implement USLE replacing climate R factor with the total event runoff (m^3^) and the event peak discharge (m^3^ s^−1^), while all other USLE factors (K, LS, C, and P) remain unchanged. This modification allows the equation to be applied to individual storm events (Arnold et al., 1998). A partial drawback of such models is their semi-distributed nature, which only allows estimating the amounts of sediment produced at watershed outlets, without providing spatially explicit results. In this regard, WaTEM/SEDEM represents a substantial difference. It is a spatially distributed sediment delivery model capable to represent erosion and deposition areas for single raster cells (and river sediment yields). Unlike SWAT and MUSLE, WaTEM/SEDEM is not a continuous-time model and can only estimate long-term average rates of annual soil loss and deposition. The soil loss is computed with the very same multi-parameter scheme generally used by USLE-type models. Subsequently, the model routes sediments downslope across each pixel from hillslopes to the riverine systems, using a transport capacity coefficient computed based on topography and land cover. A further improvement of models such as MUSLE, WaTEM/SEDEM, SWAT is the calibration of some input parameters against observed data, e.g., water discharge and sediment concentration.

Physically-based models, or process-oriented, result from more recent efforts to more comprehensively represent the complexity of soil erosion, transport, and deposition processes. They consist of algorithms derived from theoretical principles that aim to explain and predict Earth system's dynamic behavior as a whole. These models have advantages compared to empirical and semi-empirical models. For example, their ability to better represent spatiotemporal distributions of soil loss and land conditions or their transferability to a more extensive set of environments. The MMF includes aspects of both physically based and empirical models to predict annual soil loss. Like USLE-type models, MMF retains a certain level of simplicity but can also better represent the erosion process incorporating some recent developments in knowledge. The erosion process is divided into two distinct phases. First, a sediment phase which determines soil loss due to raindrop impact and runoff detachment, and a water phase that reflects the transport of soil particles by runoff. A soil erodibility factor mainly influences the detachment rate. Simultaneously, the runoff's annual transport capacity is a function of water volume, slope steepness, and a crop cover factor. WEPP is the most applied and comprehensive physically-based model listed in GASEMT. It has been structured to be applicable for an extensive range of geographic, land-use, and land-management conditions. WEPP can be applied at parcel, hillslope, road, and watershed-scale providing event or daily sediment yield, runoff, and subsurface flow. Daily rainfall conditions are described using a stochastic weather generation sub-module. The other model sub-modules describe infiltration and overland flow, erosion mechanics of sheet and rill processes, soil properties, soil tillage, and residue management, soil consolidation, and the effect of the different stages of plant growth. It uses a steady-state sediment continuity equation to describe the three steps of soil erosion (detachment, transportation, and deposition) and provides net erosion estimates. The LISEM model was one of the first physically-based models to use raster input data and a GIS environment to better account for the spatial variability of runoff and soil erosion processes within a watershed. Like WEPP, LISEM can estimate soil detachment and deposition for single events or on a continuous basis using sub-daily rainfall data to reproduce single rain events' intensity and duration. Besides describing the rainfall event, the model requires an additional set of ca. 30 input parameters to parametrize watershed conditions such as soil properties, soil surface roughness, soil moisture, saturated hydraulic conductivity vegetation cover, morphological conditions of the vegetations, among others. The RHEM model needs thirteen input parameters to represent rainfall, soils, and slope profile conditions. It was designed for rangeland application and can predict both runoff and erosion rates based on infiltration, hydrology, plant science, hydraulics, and erosion mechanics.

The insights gained in this analysis suggest that the research community is contemporaneously working on improving the application of complex process-oriented models while updating the existing empirical approaches such as the USLE, which remains attractive from a practical point of view. It also emerges that, in most cases, the selection of the soil erosion model applied is not related to the necessaty to calculate exact erosion rates for a particular situation but rather to obtain a risk estimate and compare different land conditions. These are necessities that make simple and time cost-effective soil erosion models still by far the most commonly used approaches. A development towards an increased use of physically-based models in the future remains desirable, however.

## 5 GASEMT database: data availability and limitations

All data supporting the findings of this study can be extracted from the Supplementary Information file (Appendix A: GASEMT database).

Our evaluation of the GASEMT database revealed some shortcomings. First, some missing data (reported as *unknown* in the database) are due to the unavailability of information in the reviewed publications. Although this affects only some attributes, key information such as predicted soil-erosion rates, coordinates of the study area, and the study area's size can be missing. A meta-regression analysis using GASEMT data could not be performed due to restrictions imposed by the heterogeneity of the records and, more importantly, due to a lack of detailed georeferenced information about the study areas. Due to capacity bottlenecks (our project relied on a volunteer participatory approach without funding), the scientific team refrained from defining the shape and perimeter for each study area. This situation limited the possibility of compiling variables for the modelled sites and investigating relationships between predicted erosion rates and environmental factors (e.g., climate data, slope, vegetation cover) through exploratory analysis. A further shortcoming relates to potential inconsistencies in the database. Although a harmonization procedure has been carried out to identify and rectify most inconsistencies and misclassifications among experts, some of these may still affect some records in this publication's final database. The limited number of studies compiled dealing with spatial modelling of gully, and other erosion processes (e.g., harvest erosion, tillage erosion, piping) may also have been diminished by the Scopus search criteria. Our decision to consider only research work validated/evaluated through peer-review excluded all grey literature from GASEMT, such as scientific reports, government publications, proceedings, conference abstracts, and national associations journals. We recognize that this decision could be influenced by scientific communication practices and ‘epistemic cultures’ (Cetina, 1991) that are more oriented towards peer-reviewed journals.

The exclusion of grey and non-English peer-reviewed literature in GASEMT may have affected the geographical representation of some regions in the database where publishing in local and national language/literature is more frequent. However, in light of the 419,000 results obtained searching for ‘*soil erosion modelling’* via Google Scholar, we recognize that this large amount of literature would have been an unrealistic amount to evaluate without aids such as artificial intelligence. An internal debate among the authors also highlighted whether the use of Elsevier's Scopus bibliographic database, instead of Thomson Reuters' Web of Science (WoF), or both, may have contributed to bias in the geographical distribution of the GASEMT records. The decision to use Scopus in this analysis was based on the information available in the literature, indicating that Scopus may have a greater coverage of specific subjects in Earth and Atmospheric Sciences and a larger match with compared to WoF (Barnett and Lascar, 2012; Mongeon and Paul-Hus, 2016). A recent Scopus and WoS query (17.03.2020) searching `soil erosion models` in the title, abstract, and keywords resulted in 13,474 and 12,972 articles potentially reporting applications of soil erosion modelling, respectively. Limiting the query to 1994–2017 returned similar results with 10,010 (Scopus) and 10,187 (WoS) documents. This document the similarity of Scopus and WoS and further supports the validity of our choice.

## 6 Conclusions

This systematic literature review to compile the GASEMT database and it's posterior analysis have allowed data-driven insights into the global state-of-the-art in soil erosion modelling for the first time. Statistical analysis of GASEMT showed that models tend to predict erosion rates that peak in the tropics and decrease towards higher latitudes. The frequency of model applications inversely relates to this erosion severity pattern, with greater numbers of studies in temperate and Mediterranean zones. The results of this analysis suggest that industrialised and highly developed countries, generally in temperate latitudes with lower erosion rates, show a higher incidence of studies. The database reports fewer studies in less-developed, tropical, and subtropical countries, where modelling findings suggest greater exposure to erosive processes.

Unlike previously reported work, our findings suggest that unsustainable soil-erosion rates not only occur due to a lack of policy governance (Alewell et al., 2019) but in many regions of the world may also result from a lack of knowledge. Detailed information on soil erosion, through both modelling and measurement, is lacking for large parts of the world. This condition is particularly true for regions most susceptible to high levels of soil erosion. For many of these regions we only have information from global modelling applications, which may not adequately represent local causes and drivers of soil erosion nor provide a basis for policy solutions.

Our findings indicate that (R)USLE-type models have been extensively used and modified during the last two decades and remain the most employed modelling tool today. Based on the dominance of these model types, most of our current knowledge on the spatial distribution of soil-erosion and it's temporal trends are derived through (R)USLE-type approaches. This, in turn, means that our understanding of spatial soil erosion mostly relies on empirical models dealing with water as the erosion agent and focusing on sheet and rill processes as the dominant ones. Although some models such as WEPP, RHEM, and LISEM show increasing trends of use, applications of process-based physical models appear far more constrained. Nevertheless, the scale of applications of the process-based physical models (x̃ = ~1 km^2^) suggests that the required input data are lacking for large-scale applications.

While soil erosion measurements have numerous drawbacks (eg high uncertainty, high work/time inputs, restrictions to small spatial and temporal scales), many modelling studies lack validation attempts. As such, many soil erosion modelling applications should be considered as indications of the best hypotheses currently available rather than predictive models. Unvalidated models are useful in time periods and/or locations where soil erosion measurements and monitoring data are not (yet) available. The drawbacks of unvalidated model applications means that, although numerous in the literature, they should remain transitory until better methods exist. The ease at which one can use off-the-shelf soil-erosion models using remotely sensed data and GIS has created an inflated number of models that lack field activities and validation/evaluation of the results. True knowledge gain can only be achieved by a scientific community that accepts the challenge of rethinking its approach towards modelling applications. This challenge is reflected in the UN GSERmap, which represents an opportunity to overcome some of these shortcomings by introducing new region and country-based modelling assessments supported by well-defined field-based data collection to validate/evaluate the results.

The following are the supplementary data related to this article.

Supplementary material Image 1

Appendix A GASEMT database. Appendix A

## CRediT authorship contribution statement

Pasquale Borrelli developed the research concepts, coordinated the review activities and wrote the paper. Panos Panagos and all other authors (listed in alphabetical order) contributed with ideas, reviewed the papers, worked on developing the GASEMT database, discussed the results and contributed to improving the text and the discussions of the results.

## Declaration of competing interest

The authors declare that they have no known competing financial interests or personal relationships that could have appeared to influence the work reported in this paper.
