# Supplementary material for: Soil erosion modelling: A global review and statistical analysis
Source: Sci Total Environ. 2021 Aug 1;780:146494. doi: 10.1016/j.scitotenv.2021.146494 (PMC8140410; doi:10.1016/j.scitotenv.2021.146494)
Supplement: Supplementary file 1 — Supplementary material [file mmc1.docx]

**Supplementary Information**

**Fig. S1**. Distribution of the estimated gross soil-erosion rates categorized by erosion agent (panel a), continent (panel b) and spatial scale (panel c). Values in the cells and colour legend represent the numbers of occurrence in the database.


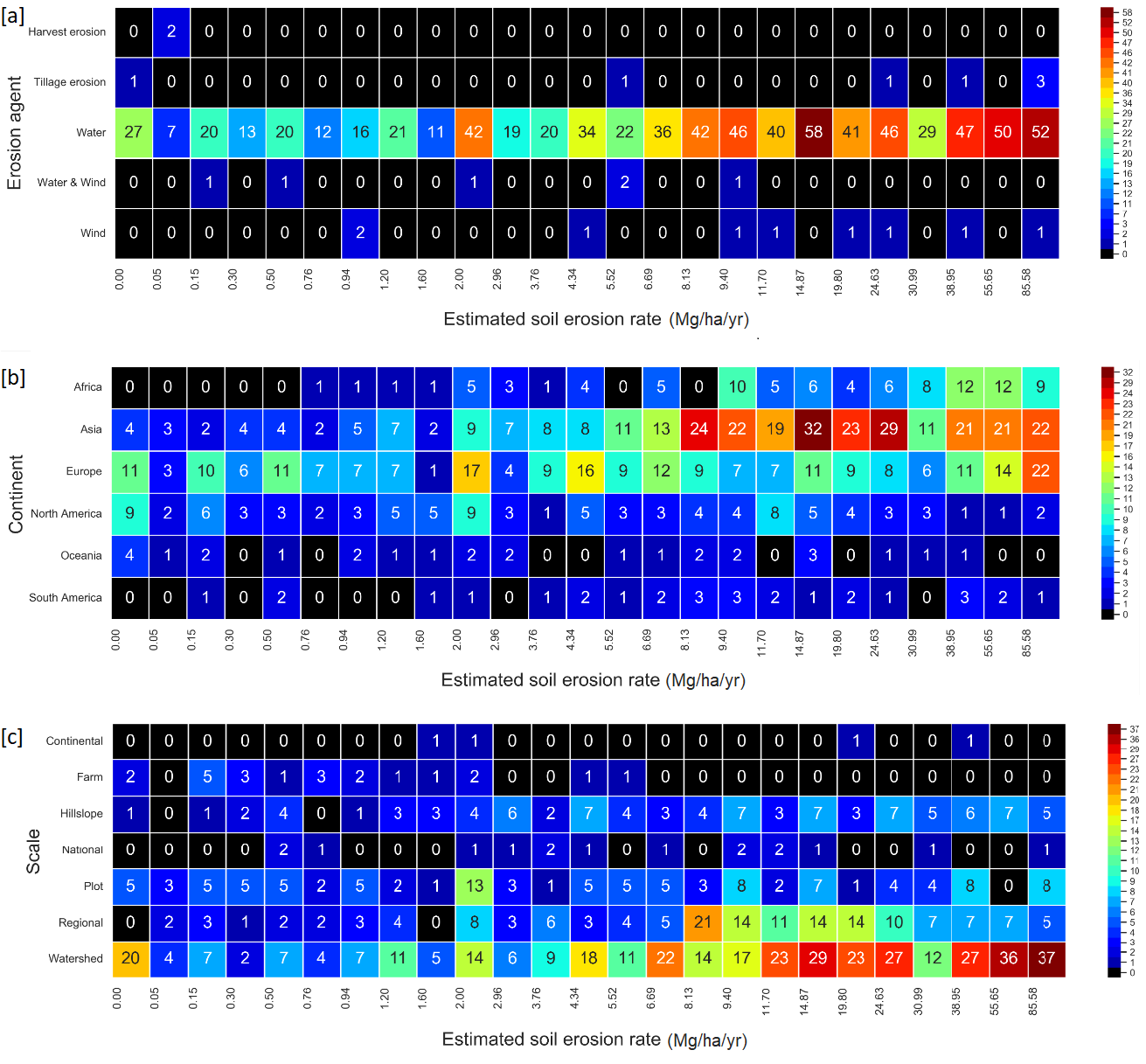


**Fig. S2**. Distribution of the estimated net soil-erosion rates (including the estimated classified in the ‘soil redistribution’ group) categorized by erosion agent (panel a), continent (panel b) and spatial scale (panel c). Values in the cells and colour legend represent the numbers of occurrence in the database.


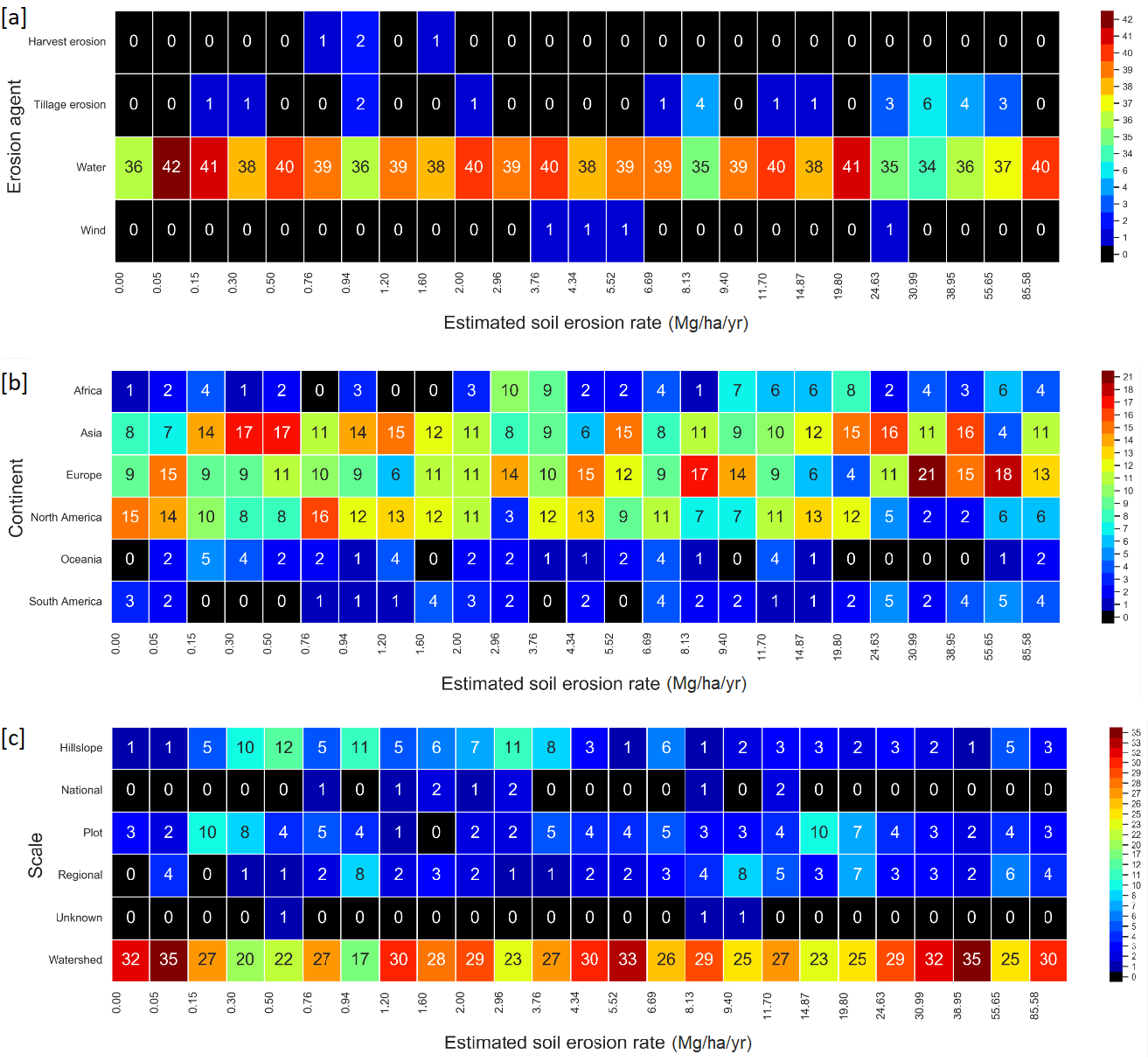


**Fig. S3**. High-resolution representation of the four panels illustrated in the Figure 10 (Robinson projection). All panels report the geographical distribution of 1,833 Global Applications of Soil Erosion Modelling Tracker (GASEMT), grouped using a hexagonal grid, superimposed on (panel a) the global cropland according to the IMAGE model year 2015 (Hurtt et al., 2019; Stehfest et al., 2014); (panel b) global annual rainfall (Hijmans et al., 2005); (panel c) global annual changes in agricultural area between the reference period 2015 and 2070 projections (Global Change Assessment Model (GCAM) RCP 6.0, Hurtt et al., 2019); and (panel d) the water and wind erosion severity according to the Global Assessment of Soil Degradation (GLASOD). In the latter case, the degree of damage is indicated from low (1) to severe (4).


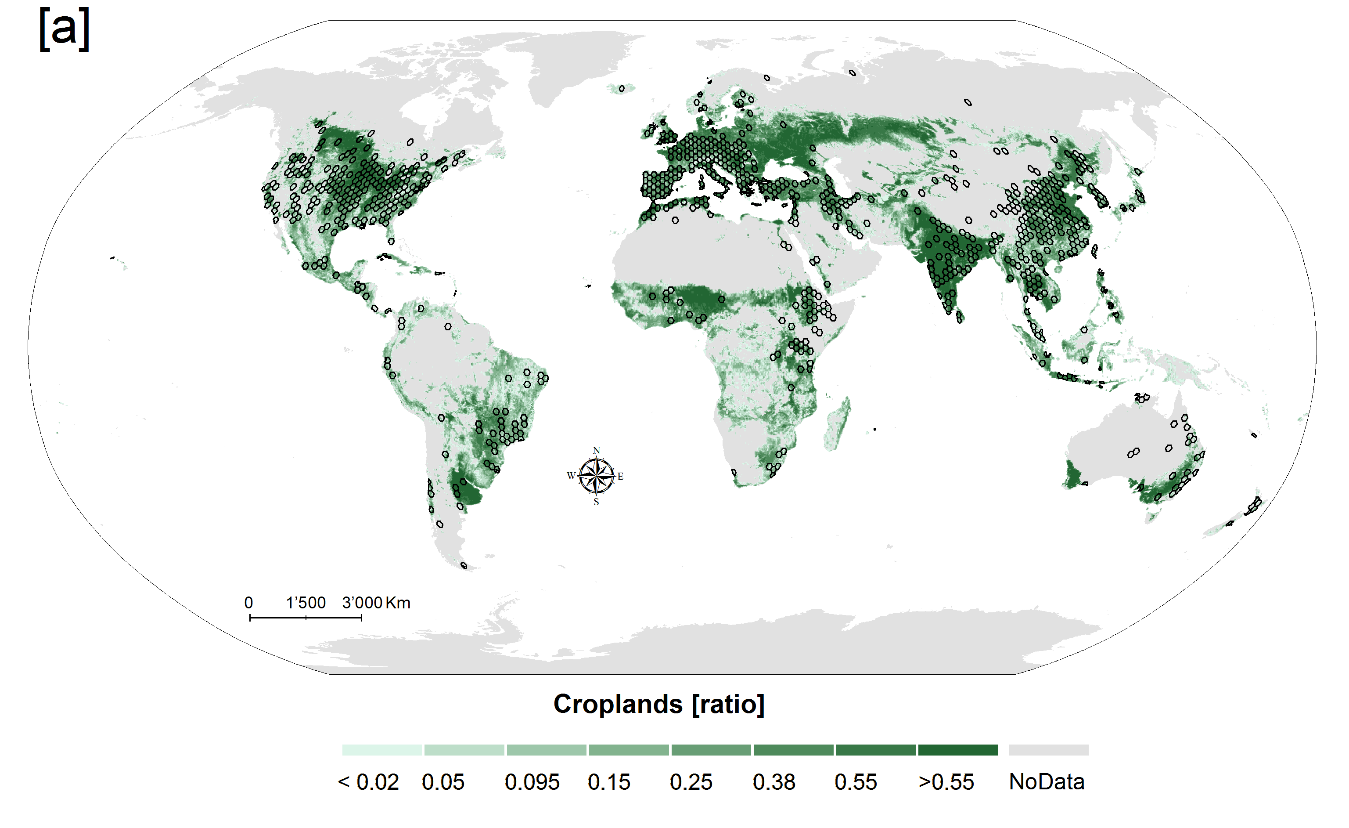


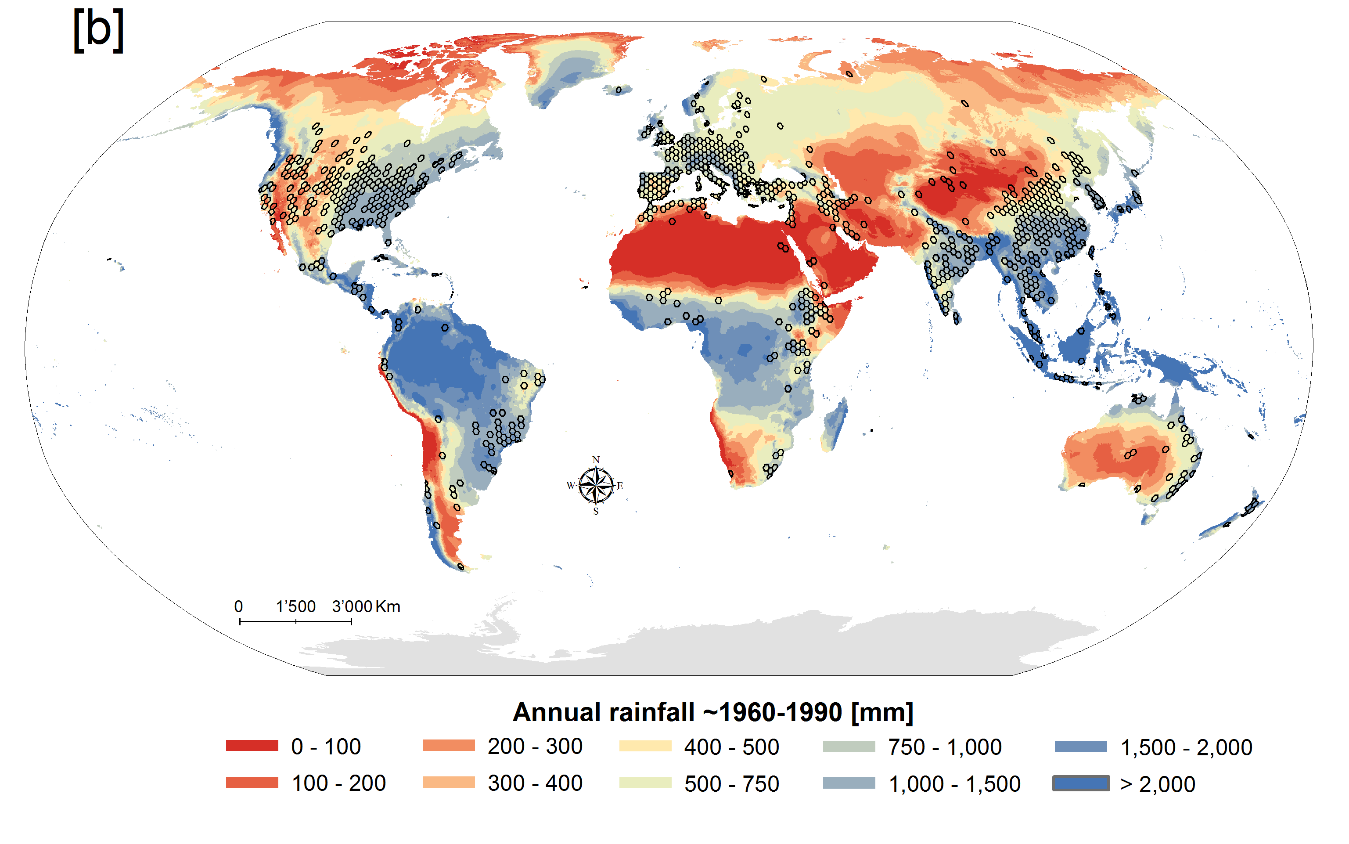


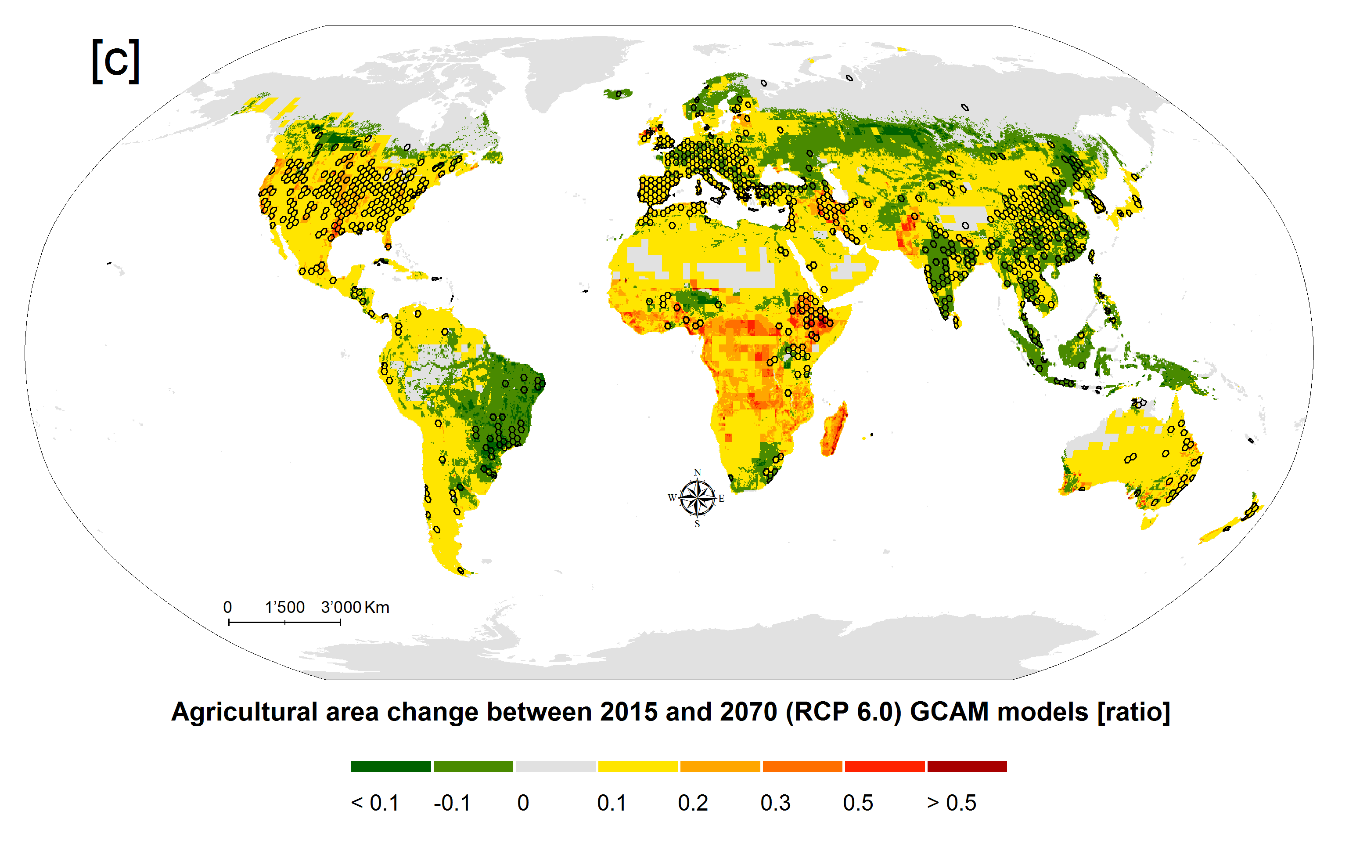


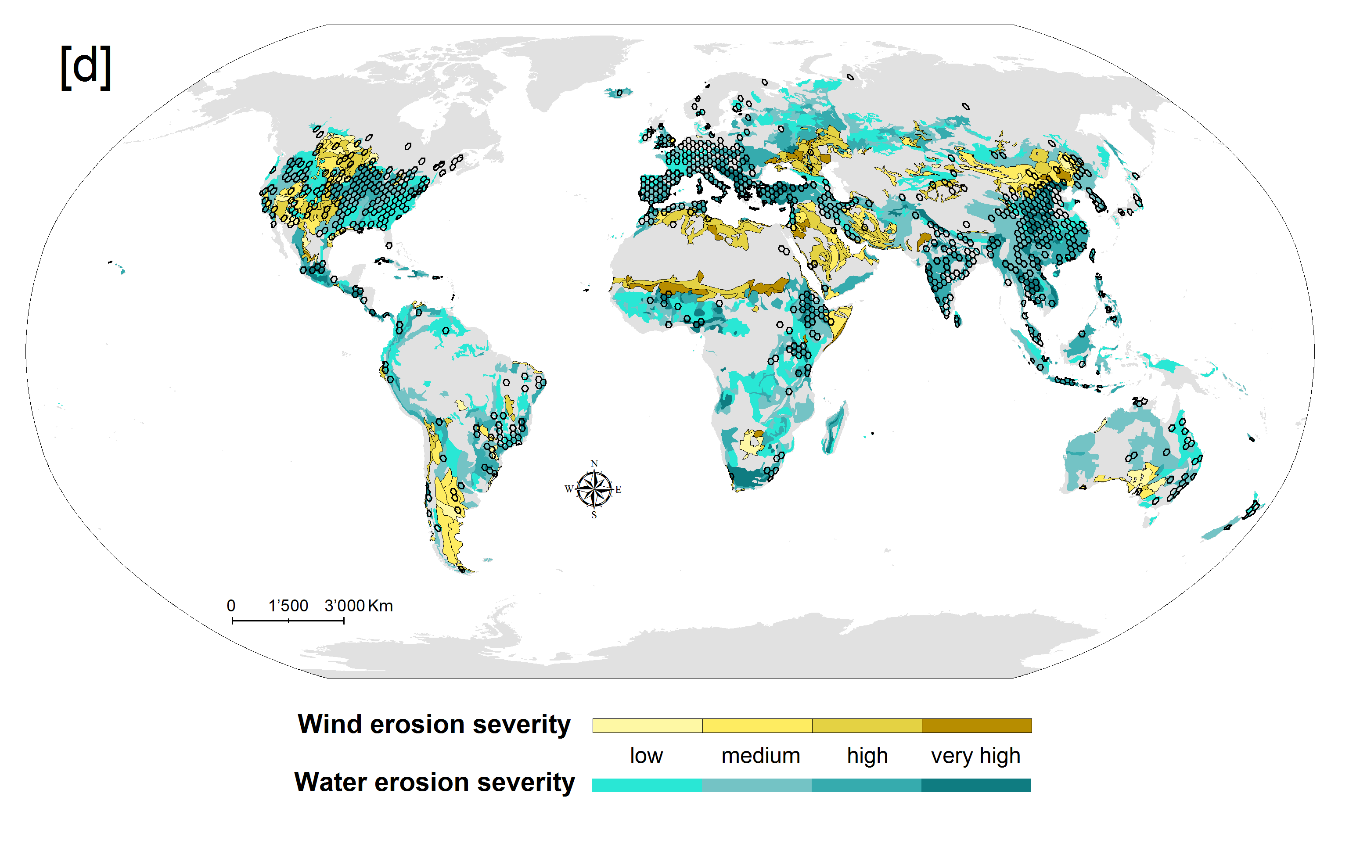


**Table S1**. Extended version of Table 1 (main text).

|  | Group | Entry | Types of data | Options |
| --- | --- | --- | --- | --- |
| i | Entry info | ID | Open (numeric) | - |
|  |  | Reviewer ID | Open (alphanumeric) | - |
|  |  | General ID | Open (alphanumeric) | - |
| ii | Bibliography | Year of publication | Open (numeric) | - |
|  |  | List of authors | Open (alphanumeric) | - |
|  |  | Title | Open (alphanumeric) | - |
|  |  | Journal | Open (alphanumeric) | - |
|  |  | DOI | Open (alphanumeric) | - |
| iii | Modelling exercise | Erosion agent | Multiple choice | Humans (harvest or tillage), water, wind, water and wind |
|  |  | Modelling type | Multiple choice | Sheet and rill, rill, gully, mass movement, stream bank, tunnel erosion, riparian erosion, sediment budget, sediment yield, sensitivity mapping, wind soil displacement, dust |
|  |  | Gross/ net estimate* | Multiple choice | Net erosion, gross erosion, soil redistribution |
|  |  | Quantitative/ qualitative estimate^$^ | Multiple choice | Quantitative, qualitative, unknown |
|  |  | Estimated soil erosion rate converted to (Mg ha^−1^ yr^−1^) | Open (numeric) | - |
|  |  | Soil erosion rate (note) | Open (alphanumeric) | - |
|  |  | Model name | Open (alphanumeric) | - |
|  |  | Modelling aim | Multiple choice | Climate change, construction site, forest harvesting, general, grazing, land use change, land use change and climate change, mining, ploughing, soil and water conservation, topographic change, unknown, wildfire |
|  |  | Modelled period | Multiple choice | Present, present & past, present & future, unknown, past, future |
| iv | Study area | Continent | Multiple choice | Asia, Oceania, Europe, Africa, North America, South America, Global, NA, unknown, North America, South-America |
|  |  | Country | Open (text) | - |
|  |  | Name of the study area | Open (alphanumeric) | - |
|  |  | Latitude (decimal degrees) | Open (numeric) | - |
|  |  | Longitude (decimal degrees) | Open (numeric) | - |
|  |  | Area (km^2^) | Open (numeric) | - |
| v | Climate | Data indicative period | Open (numeric) | - |
|  |  | Type of data | Multiple choice | Gridded, point, unknown |
|  |  | Time resolution | Multiple choice | Annual, daily, event, hourly, monthly, season, sub-hourly, unknown |
|  |  | Rainfall amount (mm) | Open (numeric) | - |
|  |  | Rainfall (note) | Open (alphanumeric) | - |
| vi | Land use/ cover | Type of data source | Multiple choice | Existing map, remote sensing & GIS mapping, field mapping, remote sensing & GIS mapping, unknown |
|  |  | Modelled area | Multiple choice | Agriculture, generic, agroforestry, all land uses, arable land, bare soil, forest, grassland/ rangeland, mines, pasture, riverbank, unknown |
| vii | Fieldwork activities | Field activities | Multiple choice | Yes, no, unknown |
|  |  | Type of activities | Multiple choice | Field mapping, field observations, general field visits, measurements, multiple activities, none, sampling, unknown |
| viii | Soil info | Soil sampling | Multiple choice | Yes, no, unknown |
|  |  | Type of soil information | Multiple choice | Point, gridded, maps, shapefile, unknown |
| ix | Topography | DEM cell size (m) | Open (numeric) | - |
| x | Modelling outcomes | Scale^§^ | Multiple choice | Continental, farm, global, hillslope, national, plot, regional, unknown, watershed (understood as hydrologic unit) |
|  |  | Cell size (m) | Open (numeric) | - |
|  |  | Modelled years | Open (numeric) | - |
|  |  | Modelled period | Multiple choice | ≤2000, >2000, both periods, unknown |
|  |  | Validation/evaluation attempt of model results | Multiple choice | Yes, no, unknown |
|  |  | Type of validation/evaluation | Multiple choice | Comparisons with results from other models, expert knowledge, measured erosion rates, measured SY, NA, unknown |
|  |  | Model calibration | Multiple choice | Yes, no, NA, unknown |

**Table S2**. Scale definition.

| **Scale** | **Definition** |
| --- | --- |
| Continental | encompassing the total area within the boundaries of a continent |
| Farm | Agricultural parcel |
| Global | Terrestrial surface of the earth |
| Hillslope | Plot that extends towards the upper topographic boundary of a slope |
| National | Encompassing the total area within the boundaries of a nation |
| Plot | Defined area within a slope |
| Regional | Larger than watershed, smaller than national |
| Watershed | Surface area from which runoff resulting from rainfall is collected and drained through a common point. It is synonymous with a drainage basin or catchment area |

**References**

Hijmans, R.J., Cameron, S.E., Parra, J.L., Jones, P.G., Jarvis, A., 2005. Very high resolution interpolated climate surfaces for global land areas. Int. J. Climatol. 15, 1965–1978. https://doi.org/10.1002/joc.1276

Hurtt, G., Chini, L., Sahajpal, R., Frolking, S., Al., E., 2019. Harmonization of global land-use change and management for the period 850-2100. http://luh.umd.edu/ (in preparation).

Stehfest, E., van Vuuren, D., Kram, T., Bouwman, L., Alkemade, R., Bakkenes, M., Biemans, H., Bouwman, A., Den Elzen, M., Janse, J., Alkemade, R., Bakkenes, M., Biemans, H., Bouwman, A., Den Elzen, M., Janse, J., Lucas, P., van Minnen, J., Muller, C., Prins, A.G., 2014. Integrated assessment of global environmental change with IMAGE 3.0, Model description and policy applications, The Hague: PBL Netherlands Environmental Assessment Agency.
